# Convergent synthesis of the pentasaccharide repeating unit of the biofilms produced by *Klebsiella pneumoniae*

**DOI:** 10.3762/bjoc.15.37

**Published:** 2019-02-13

**Authors:** Arin Gucchait, Angana Ghosh, Anup Kumar Misra

**Affiliations:** 1 Bose Institute, Division of Molecular Medicine, P-1/12, C.I.T. Scheme VII M, Kolkata 700054, India

**Keywords:** beta-D-mannoside, biofilms, D-glucuronic acid, glycosylation, *Klebsiella pneumoniae*, pentasaccharide, polysaccharide

## Abstract

A pentasaccharide repeating unit containing α-linked D-glucuronic acid, β-linked D-mannose, corresponding to the repeating unit of biofilms produced by *Klebsiella pneumoniae,* has been synthesized using a stereoselective [2 + 3] convergent glycosylation strategy. The β-D-mannosidic moiety has been synthesized using a D-mannose-derived thioglycoside by a two-step activation process. Late stage TEMPO-mediated oxidation of the pentasaccharide derivative using phase-transfer reaction conditions furnished the target compound in satisfactory yield.

## Introduction

*Klebsiella pneumoniae* (*K. pneumoniae*) is a Gram-negative pathogenic organism causing pneumonia, urinary tract infections (UTI), intra-abdominal infections, meningitis, and pyrogenic liver abscesses in humans [1–3]. The bacteria have capsules in the outermost layers of the cells, which are composed of a variety of complex polysaccharides known as K-antigens [4]. The pathogenicity of *K. pneumoniae* relies on the structure of the capsular polysaccharides [5]. Besides a large number of polysaccharides present in the capsules, *K. pneumoniae* secrets a variety of polysaccharides which form biofilms for the protection of the organism from external agents [6]. In addition, secreted polysaccharides from a particular strain also render pathogenicity of that organism. Since capsular polysaccharides of *K. pneumoniae* and polysaccharides secreted by it play an important role in the initial stage of infections [7], it is quite pertinent to develop therapeutics based on the glycoconjugate derivatives of these polysaccharides. Isolation of polysaccharides by fermentation of bacterial strains suffer from a number of limitations, which include (a) handling of live strains of bacteria; (b) difficulties in separating biological impurities such as proteins or nucleic acids and (c) loss of homogeneity of the polysaccharide fragments to name a few. It has already been established that in many cases the oligosaccharide repeating units resemble similar antigenic potential to those of the native polysaccharides [8–10]. Therefore, the best alternative way to get the oligosaccharide fragments corresponding to the capsular polysaccharides or polysaccharides secreted by the bacterial strains, is to develop effective synthetic strategies. The structure and glycosyl linkages of the monosaccharide units remain conserved in the synthesized oligosaccharides in contrast to the natural version. The synthesized molecules can be linked with appropriate functionalities for their conjugation with a protein for the preparation of glycoconjugates. Recently, Cescutti et al. [11] reported the structure of a pentasaccharide composed of D-glucose, D-mannose and D-glucuronic acid corresponding to the repeating unit of the biofilm producing polysaccharide secreted by *K. pneumoniae*. The unique feature of this pentasaccharide is that it contains a β-D-mannosidic moiety and a α-D-glucuronic acid moiety, which are known to be challenging from the synthetic point of view. In addition, two α-linked D-mannose moieties and one β-linked D-glucose unit are present in the molecule. In view of the importance of oligosaccharides in the development of glycoconjugate-based therapeutics, it was decided to undertake the synthesis of a pentasaccharide corresponding to the repeating unit of the biofilm producing polysaccharide secreted by *K. pneumoniae*. A convergent synthesis of a pentasaccharide as its 2-aminoethyl glycoside containing a β-D-mannosidic moiety and a α-D-glucuronic acid moiety is presented herein. The presence of a 2-aminoethyl group at the reducing end of the pentasaccharide may provide ready availability of an amino functionality to expedite the conjugation of the pentasaccharide with an appropriate protein without affecting the sugar rings in the molecule [12–13].

## Results and Discussion

**Figure 1:**
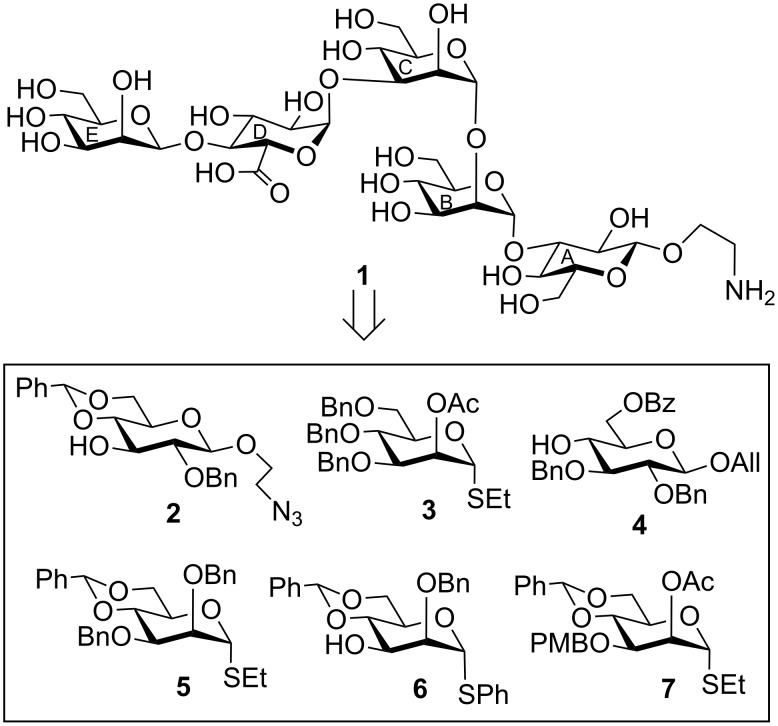
Structure of the synthesized pentasaccharide corresponding to the repeating unit of the biofilms produced by *Klebsiella pneumoniae.*

The target pentasaccharide as its 2-aminoethyl glycoside **1** was synthesized using a set of stereoselective glycosylations of a number of suitably functionalized monosaccharide derivatives **2**, **3** [14], **4**, **5** [15], **6** [16] and **7** [17], which were prepared from the commercially available reducing monosaccharides using a number of functional group manipulations reported earlier (Figure 1).

Initially it was planned to couple the disaccharide acceptor **13** with the trisaccharide thioglycoside donor **19** using a [3 + 2] convergent glycosylation strategy to achieve the pentasaccharide derivative **20**. However, the desired product was obtained in poor yield, which did not allow upscaling of the synthesis. Consequently, an alternative [2 + 3] block glycosylation strategy was adopted using the disaccharide trichloroacetimidate derivative **18** as donor and trisaccharide derivative **23** as glycosyl acceptor, which resulted in the formation of target pentasaccharide derivative **20** in satisfactory yield with excellent stereoselectivity. The noteworthy features of the synthetic strategy are (a) incorporation of a β-D-mannosidic linkage and (b) late stage TEMPO-mediated oxidation of the primary hydroxy group into a carboxylic group after completion of glycosylations.

**Scheme 1:**
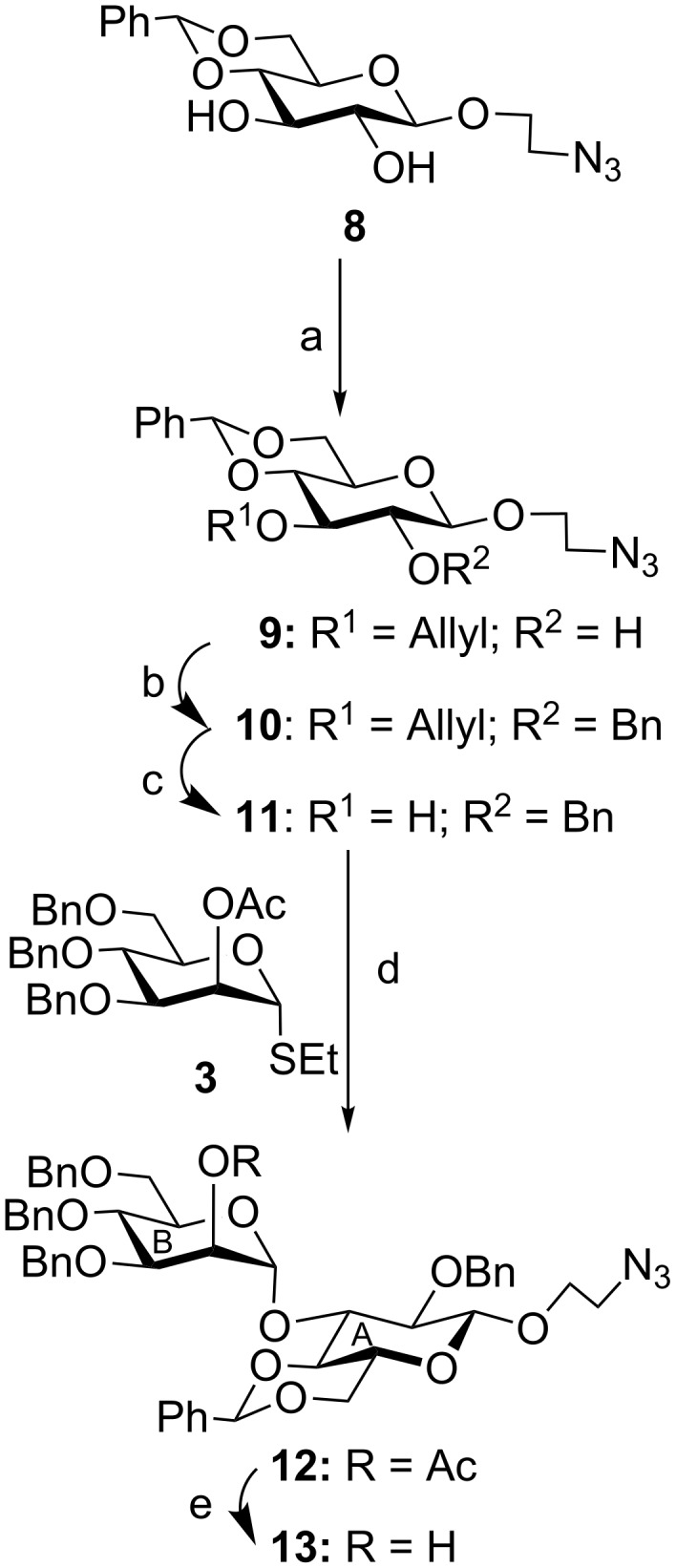
Reagents and conditions: (a) i: Bu_2_SnO, CH_3_OH, 80 °C, 2 h; ii: allyl bromide, CsF, DMF, 65 °C, 6 h; (b) benzyl bromide, NaH, DMF, 0 °C, 1 h, 90% in two steps; (c) PdCl_2_, CH_3_OH, 2 h, 70%; (d) NIS, TMSOTf, –10 °C, 30 min, 80%; (e) 0.1 M CH_3_ONa in CH_3_OH, room temperature, 2 h, 98%.

The starting compound 2-azidoethyl 4,6-*O*-benzylidene-β-D-glucopyranoside (**8**) [18], prepared from D-glucose, was selectively *O*-allylated at the 3-hydroxy group by treatment with dibutyltin oxide followed by allyl bromide in the presence of cesium fluoride [19] via the formation of a stannylene acetal to give 2-azidoethyl 3-*O*-allyl-4,6-*O*-benzylidene-β-D-glucopyranoside (**9**). Compound **9** was treated with benzyl bromide in the presence of sodium hydride [20] to give *O*-benzylated derivative **10** in 90% yield in two steps, which on de-*O*-allylation by treatment with palladium chloride [21] furnished 2-azidoethyl 2-*O*-benzyl-4,6-*O*-benzylidene-β-D-glucopyranoside (**11**) in 70% yield. Stereoselective glycosylation of compound **11** with D-mannose-derived ethyl 2-*O*-acetyl-3,4,6-tri-*O*-benzyl-1-thio-α-D-mannopyranoside (**3**) [14] in the presence of a combination of *N*-iodosuccinimide (NIS) and trimethylsilyl trifluoromethanesulfonate (TMSOTf) [22–23] exclusively furnished 2-azidoethyl (2-*O*-acetyl-3,4,6-tri-*O*-benzyl-1-thio-α-D-mannopyranosyl)-(1→3)-2-*O*-benzyl-4,6-*O*-benzylidene-β-D-glucopyranoside (**12**) in 80% yield, which on treatment with sodium methoxide [24] led to the formation of disaccharide acceptor **13** in 98% yield (Scheme 1).

**Scheme 2:**
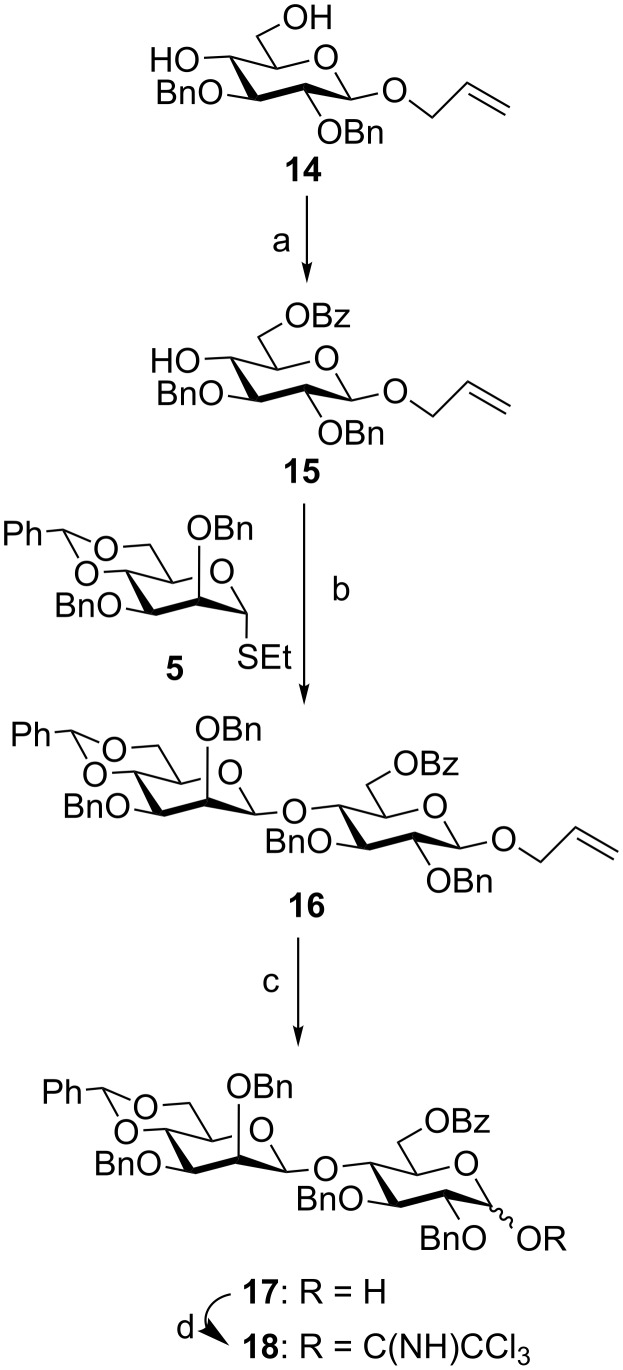
Reagents and conditions: (a) Benzoyl chloride, pyridine, 0 °C, 3 h, 75%; (b) Tf_2_O, BSP, TTBP, CH_2_Cl_2_, MS 4 Å, −60 °C, 1 h then compound **10**, −78 °C, 2 h, 65%; (c) PdCl_2_, CH_3_OH, 0 °C to room temperature, 3 h, 75%; (d) CCl_3_CN, DBU, CH_2_Cl_2_, −10 °C, 90% (mixture of α and β).

In another experiment, allyl 2,3-di-*O*-benzyl-β-D-glucopyranoside (**14**) [25], prepared from D-glucose, was selectively 6-*O*-benzoylated by treatment with benzoyl chloride and pyridine at low temperature [26] to give allyl 6-*O*-benzoyl-2,3-di-*O*-benzyl-β-D-glucopyranoside (**15**) in 75% yield. The next step was to synthesize the 1,2-*cis* glycosylated product of the D-mannose moiety (β-mannoside), which was considered as a challenging task in spite of several reaction strategies developed in the recent past [16,27–33]. Among several elegant reaction conditions, it was decided to apply a two-step activation of the benzylidene-thiomannoside donor in the presence of a combination of 1-benzenesulfinylpiperidine (BSP) and 2,4,6-tri-*tert*-butylpyrimidine (TTBP) and then triflic anhydride (Tf_2_O) developed by Crich et al. [28–29]. Stereoselective 1,2-*cis* glycosylation of D-mannose-derived ethyl 4,6-*O*-benzylidene-2,3-di-*O*-benzyl-1-thio-α-D-mannopyranoside (**5**) [15] with compound **15** under a two-step activation using a combination of BSP and triflic anhydride in the presence TTBP as a base furnished allyl (4,6-*O*-benzylidene-2,3-di-*O*-benzyl-β-D-mannopyranosyl)-(1→4)-6-*O*-benzoyl-2,3-di-*O*-benzyl-β-D-glucopyranoside (**16**) in 65% yield together with some 1,2-*trans* glycosylated product (≈10%), which was separated by column chromatography. Compound **16** was treated with palladium chloride [21] to give the disaccharide hemiacetal derivative **17** in 75% yield, which on treatment with trichloroacetonitrile in the presence of DBU [34] resulted in the formation of (4,6-*O*-benzylidene-2,3-di-*O*-benzyl-β-D-mannopyranosyl)-(1→4)-6-*O*-benzoyl-2,3-di-*O*-benzyl-α,β-D-glucopyranosyl trichloroacetimidate (**18**) in 90% yield (Scheme 2).

**Scheme 3:**
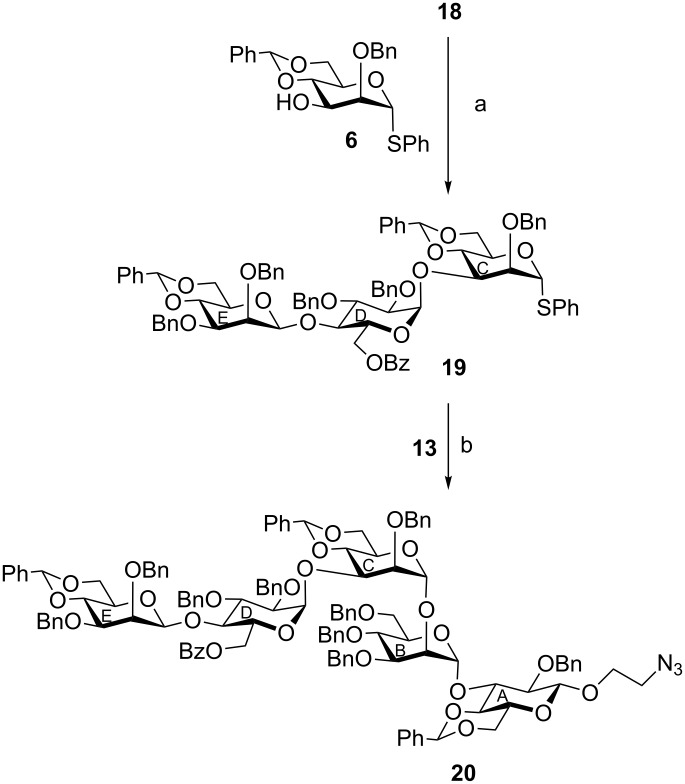
Reagents and conditions: (a) TMSOTf, CH_2_Cl_2_, −10 °C, 30 min, 45%; (b) NIS, TMSOTf, MS 4 Å, CH_2_Cl_2_, −10 °C, 30 min, 40%.

In the next set of reactions, the disaccharide trichloroacetimidate derivative **18** was allowed to couple with the D-mannose-derived thioglycoside acceptor **6** in the presence of TMSOTf [35] to furnish phenyl (4,6-*O*-benzylidene-2,3-di-*O*-benzyl-β-D-mannopyranosyl)-(1→4)-(6-*O*-benzoyl-2,3-di-*O*-benzyl-α-D-glucopyranosyl)-(1→3)-4,6-*O*-benzylidene-2-*O*-benzyl-1-thio-α-D-mannopyranoside (**19**) in 45% yield. The trisaccharide thioglycoside **19** can now act as a glycosyl donor fulfilling the orthogonal glycosylation principle [36]. Stereoselective glycosylation of disaccharide acceptor **13** with the trisaccharide donor **19** in the presence of a combination of NIS and TMSOTf [22–23] resulted in the formation of pentasaccharide derivative **20** in 40% yield (Scheme 3).

**Scheme 4:**
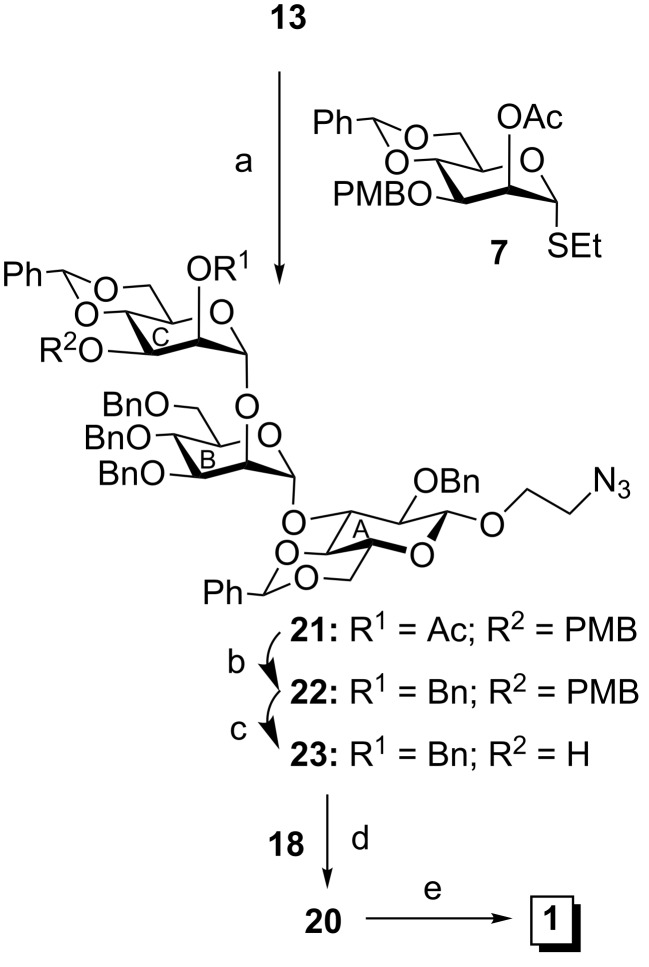
Reagents and conditions: (a) NIS, TMSOTf, MS 4 Å, CH_2_Cl_2_, −50 °C, 2 h, 70%; (b) benzyl bromide, NaOH, TBAB, DMF, 3 h; (c) DDQ, CH_2_Cl_2_/H_2_O, room temperature, 84%; (d) TMSOTf, CH_2_Cl_2_, −10 °C, 30 min, 70%; (e) i: 0.1 M CH_3_ONa in CH_3_OH, room temperature, 4 h; ii: TEMPO, NaOCl, NaBr, TBAB, NaHCO_3_, 5 °C, 3 h then NaClO_2_, NaH_2_PO_4_, *tert*-butanol, 2-methylbut-2-ene, room temperature, 3 h; iii: 20% Pd(OH)_2_/C, H_2_, CH_3_OH, room temperature, 24 h, over all 61%.

Although the pentasaccharide derivative **20** was obtained, the yield was significantly poor. Therefore, it was decided to modify the synthetic strategy to improve the yield of pentasaccharide derivative **20** and a [2 + 3] block glycosylation strategy has been designed. Compound **13** was α-selectively glycosylated with D-mannose-derived thioglycoside donor **7** in the presence of a combination of NIS and TMSOTf to furnish trisaccharide derivative **21** in 70% yield. Compound **21** was transformed into the trisaccharide acceptor **23** via a two-step reaction sequence involving removal of acetyl groups and benzylation using benzyl bromide and sodium hydroxide in one-pot [37] followed by removal of the *p*-methoxybenzyl group using DDQ [38] in 84% overall yield. Finally, stereoselective glycosylation of disaccharide trichloroacetimidate donor **18** with trisaccharide acceptor **23** in the presence of TMSOTf [35] furnished target pentasaccharide derivative **20** in 70% yield (Scheme 4). Removal of the benzoyl group from compound **20** using sodium methoxide [24] followed by TEMPO-mediated oxidation [39] of the hydroxy group to a carboxylic group using sodium hypochlorite under biphasic reaction conditions furnished the D-glucuronic acid containing pentasaccharide derivative, which on global deprotection under hydrogenolysis in the presence of Pearlman’s catalyst [40] afforded the target pentasaccharide as its 2-aminoethyl glycoside **1** in 61% yield (Scheme 4).

## Conclusion

In summary, a convergent synthesis of a pentasaccharide corresponding to the repeating unit of the biofilm producing polysaccharide secreted by *K. pneumoniae* as its 2-aminoethyl glycoside has been achieved in good yield. The noteworthy points of the synthetic strategy include stereoselective construction of a β-D-mannosidic glycosyl bond and late stage oxidation of a primary hydroxy group into a carboxylic acid. All intermediate steps were high yielding and the stereochemical outcomes of the glycosylation reactions were excellent.

## Supporting Information

File 1 Experimental procedures and NMR spectra.
