# Supplementary material for: Convergent synthesis of the pentasaccharide repeating unit of the biofilms produced by Klebsiella pneumoniae
Source: Beilstein J Org Chem. 2019 Feb 13;15:431–6. doi: 10.3762/bjoc.15.37 (PMC6404407; doi:10.3762/bjoc.15.37)
Supplement: File 1 — Experimental procedures and NMR spectra. [file Beilstein_J_Org_Chem-15-431-s001.pdf]

BEILSTEIN JOURNAL OF ORGANIC CHEMISTRY

Supporting Information

for

Convergent synthesis of the pentasaccharide repeating unit of the biofilms produced by *Klebsiella pneumoniae*

Arin Gucchait, Angana Ghosh and Anup Kumar Misra

Beilstein J. Org. Chem. **2019,** *15,* 431–436. doi:10.3762/bjoc.15.37

Experimental procedures and NMR spectra

License and Terms: This is a supporting information file under the terms of the Creative Commons Attribution License (http://creativecommons.org/licenses/by/4.0). Please note that the reuse, redistribution and reproduction in particular requires that the authors and source are credited.
The license is subject to the *Beilstein Journal of Organic Chemistry* terms and conditions: (https://www.beilstein-journals.org/bjoc)

Index

Subject	Page No.
Detailed experimental procedure for the synthesized compounds	S**2–S11**
1D and 2D NMR spectra of compounds **1, 10, 11, 12, 13, 15, 16, 19, 20, 21, 22** and **23**	**S12–S29**

Experimental

General methods

All reactions were monitored by thin layer chromatography over silica gel coated TLC plates. Silica gel 230–400 mesh was used for column chromatography. ^{1}H and ^{13}C NMR spectra were recorded on a Bruker Avance 500 MHz spectrometer using $CDCl_3$ as solvent and TMS as the internal reference unless stated otherwise. Chemical shift values are expressed in δ ppm. ESI-MS were recorded on a Micromass mass spectrometer. Elementary analysis was carried out on a Carlo Erba analyzer. Commercially available grades of organic solvents of adequate purity were used in many reactions.

2-Azidoethyl 3-*O*-allyl-2-*O*-benzyl-4,6-*O*-benzylidene-β-D-glucopyranoside (10): A suspension of compound **8** (2 g; 5.93 mmol) and Bu_2SnO (1.77 g; 7.1 mmol) in CH_3OH (50 mL) was allowed to stir at 80 °C for 2 h. The solvents were removed under reduced pressure and co-evaporated with toluene (3 × 50 mL). The colourless foam was dried using reduced pressure for 2 h and dissolved in anhydr. DMF (30 mL). To the solution were added CsF (900 mg; 5.93 mmol) and allyl bromide (515 µL; 5.93 mmol) and the reaction mixture was allowed to stir at 65 °C for 6 h. The solvents were evaporated and the crude mass was diluted with EtOAc (100 mL) and washed with brine (100 mL). The organic layer was separated, dried (Na_2SO_4) and evaporated to give compound **9**. To a solution of compound **9** (1.4 g; 3.71 mmol) in anhydrous DMF (15 mL) were added NaH (178 mg; 7.4 mmol) and BnBr (0.48 mL; 4.08 mmol) at 0 °C and the reaction mixture was stirred at same temperature for 1 h. The reaction was quenched with aq. NH_4Cl (50 mL) and diluted with CH_2Cl_2 (50 mL). The organic layer was separated, dried (Na_2SO_4) and evaporated to dryness. The crude residue was purified over SiO_2 (30% EtOAc/hexane) to give pure compound **10** (1.56 g, 90%) as colourless oil. $[\alpha]_D^{25}$ +64 (*c* 1.0, $CHCl_3$); IR (neat): 3360, 2933, 2145, 1452, 1233, 1086, 759 cm^{-1}; ^{1}H NMR (500 MHz, $CDCl_3$): δ 7.47-7.25 (m, 10H, Ar-*H*), 5.98-5.88 (m, 1 H, C*H*=CH_2), 5.53 (br s, 1 H, PhC*H*), 5.28-5.13 (m, 2 H, CH=C*H*$_2$), 4.88 (d, *J* = 11 Hz, 1 H, PhC*H*), 4.79 (d, *J* = 11.0 Hz, 1 H, PhC*H*), 4.50 (d, *J* = 8.0 Hz, 1 H, H-1), 4.38-4.36 (m, 1 H, OC*H*$_2$-CH=CH_2), 4.33 (dd, *J* = 10.5 Hz, 5.0 Hz, 1 H, H-6_a), 4.27- 4.25 (m, 1 H, OC*H*$_2$-CH=CH_2), 4.03-4.01 (m, 1 H, H-5), 3.78 (t, *J* = 10.5 Hz each, 1 H, H-6_b), 3.74-3.71 (m, 1 H, OC*H*), 3.62-3.60 (m, 2 H, H-2, H-3), 3.47-3.47 (m, 4 H, H-4, OC*H*, NC*H*$_2$); ^{13}C NMR (125 MHz, $CDCl_3$): δ 138.3-126 (Ar-C), 116.9 (CH=*C*H$_2$), 103.9 (C-1), 101.1 (Ph*C*H), 81.9 (C-5),

81.3 (C-3), 80.5 (C-4), 75.4 (Ph*C*H_2), 74.0 (O*C*H_2-CH=CH_2), 68.5 (C-6, O*C*H_2), 50.9 (N*C*H_2); ESI-MS: 490.2 $[M+Na]^+$; Anal. Calcd. for $C_{25}H_{29}N_3O_6$ (467.51): C, 64.23; H, 6.25; found: C, 64.10; H, 6.40.

2-Azidoethyl 2-*O*-benzyl-4,6-*O*-benzylidene-β-D-glucopyranoside (11): To a solution of compound **10** (1.5 g; 3.21 mmol) in anhydrous CH_3OH (15 ml) was added $PdCl_2$ (115 mg; 0.64 mmol) at 0 °C and the reaction was continued at same temperature for 2 h and at room temperature for another 2 h. The reaction mixture was filtered through a Celite bed and the filtrate was concentrated under reduced pressure. The crude product was purified over SiO_2 (40% EtOAc/hexane) to give pure compound **11** (960 mg, 70%) as white foam. $[\alpha]_D^{25}$ +123 (*c* 1.0, $CHCl_3$); IR (neat): 3356, 2930, 2150, 1444, 1230, 697 cm^{-1}; ^1H NMR (500 MHz, $CDCl_3$): δ 7.46-7.26 (m, 10 H, Ar-H), 5.46 (br s, 1 H, PhC*H*), 4.91 (d, *J* = 11.0 Hz, 1 H, PhC*H*), 4.73 (d, *J* = 11.0 Hz, 1 H, PhC*H*), 4.45 (d, *J* = 8.0 Hz, 1 H, H-1), 4.27 (dd, *J* = 10.5 Hz, 5.0 Hz, 1 H, H-6_a), 3.99-3.95 (m, 1 H, OC*H*), 3.75 (t, *J* = 9.0 Hz each, 1 H, H-4), 3.70 (t, *J* = 10.5 Hz each, 1 H, H-6_b), 3.68-3.65 (m, 1 H, OC*H*), 3.46 (t, *J* = 10.5 Hz, 1 H, H-3), 3.44-3.42 (m, 2 H, NCH_2), 3.35 (t, *J* = 8.5 Hz each, 1 H, H-2), 2.7 (br s, 1 H, O*H*); ^{13}C NMR (125 MHz, $CDCl_3$): δ 138.3-126.3 (Ar-C), 103.7 (C-1), 101.7 (Ph*C*H), 81.9 (C-5), 80.3 (C-3), 74.9 (C-4), 73.1 (Ph*C*H_2), 68.6 (C-6), 68.4 (O*C*H_2), 60.3 (C-2), 50.9 (N*C*H_2); ESI-MS: 450.1 $[M+Na]^+$; Anal. Calcd. for $C_{22}H_{25}N_3O_6$ (427.45): C, 61.82; H, 5.90; found: C, 61.65; H, 6.10.

2-Azidoethyl (2-*O*-acetyl-3,4,6-tri-*O*-benzyl-α-D-mannopyranosyl)-(1→3)-2-*O*-benzyl-4,6-*O*-benzylidene-β-D-glucopyranoside (12): In a similar manner to a procedure reported earlier [1], to a solution of compound **11** (0.9 g; 2.11 mmol) and glycosyl donor **3** (1.6 g; 2.74 mmol) in dry CH_2Cl_2 (20 mL) was added MS 4 Å (1 g) and allowed to stir at room temperature under argon for 30 min. To the reaction mixture was added NIS (680 mg; 3.01 mmol) and it was cooled to −10 °C under argon. To the cooled reaction mixture was added TMSOTf (50 µL) and it was allowed to stir at same temperature for 30 min. The reaction mixture was diluted with CH_2Cl_2 (50 mL) and the organic layer was washed with 5% $Na_2S_2O_3$ (50 mL), aq. satd. $NaHCO_3$ (50 mL) and water (50 mL). The organic layer was separated, dried (Na_2SO_4) and evaporated to dryness. The crude residue was purified over SiO_2 (50% EtOAc/hexane) to give pure compound **12** (1.52 g, 80%) as a colourless oil. $[\alpha]_D^{25}$ −18 (*c* 1.0, $CHCl_3$); IR (neat): 3260, 3031, 2930, 2155, 1760, 1388, 1112, 769 cm^{-1}; ^1H NMR (500 MHz, $CDCl_3$): δ 7.40-7.05 (m, 25 H, Ar-H), 5.54 (br s, 1 H, PhC*H*), 5.49 (br s, 1 H, H-2_B), 5.36 (br s, 1 H, H-1_B), 4.86 (d, *J* = 10.5 Hz, 1 H, PhC*H*), 4.81 (d, *J* = 10.0 Hz, 1 H,

PhCH), 4.70 (d, J = 11.0 Hz, 1 H, PhCH), 4.58-4.50 (m, 3 H, 3 PhCH), 4.49 (d, J = 8.0 Hz, 1 H, H-1_A), 4.43 (d, J = 11.0 Hz, 1 H, PhCH), 4.35 (m, 1 H, H-6_{aA}), 4.25 (d, J = 12.0 Hz, 1 H, PhCH), 4.01-3.95 (m, 2 H, H-5_A, H-6_{bA}), 3.93-3.91 (m, 3 H, H-3_B, H-4_B, H-5_B), 3.79-3.74 (m, 2 H, OCH_2), 3.63 (t, J = 9.0 Hz each, 1 H, H-4_A), 3.48-3.35 (m, 3 H, H-6_{aB}, NCH_2), 3.34-3.32 (m, 3 H, H-2_A, H-3_A, H-6_{bB}), 2.90 (br s, 3 H, COCH_3); ^{13}C NMR (125 MHz, $CDCl_3$): δ 169 (COCH$_3$), 137.8-124.9 (Ar-C), 103.1 (C-1_A), 101.0 (PhCH), 97.7 (C-1_B), 81.7 (C-4_A), 80.2 (C-3_A), 77.8 (C-4_B), 75.5 (PhCH$_2$), 75.2 (PhCH$_2$), 75.1 (C-3_B), 74.2 (C-5_B), 73.1 (PhCH$_2$), 71.5 (PhCH$_2$), 71.1 (C-5_A), 68.6 (OCH$_2$), 68.5 (C-6_B), 68.4 (C-6_A), 68.3 (C-2_B), 65.7 (C-2_A), 50.9 (NCH$_2$), 21.0 (COCH$_3$); MALDI MS: 924.3 [M+Na]$^+$; Anal. Calcd. for $C_{51}H_{55}N_3O_{12}$ (901.99): C, 67.91; H, 6.15; found: C, 67.75; H, 6.30.

2-Azidoethyl (3,4,6-tri-*O*-benzyl-α-D-mannopyranosyl)-(1→3)-2-*O*-benzyl-4,6-*O*-benzylidene-β-D-glucopyranoside (13): A solution of compound **12** (1.5 g; 1.66 mmol) in 0.1 M CH_3ONa in CH_3OH (20 mL) was stirred at room temperature for 2 h and neutralized by adding Dowex 50W X8 (H$^+$) resin. The reaction mixture was filtered and the filtrate was evaporated to dryness. The crude residue was purified over SiO_2 (50% EtOAc/hexane) to give pure compound **13** (1.4 g, 98%) as a white foam. $[\alpha]_D^{25}$ −12 (c 1.0, $CHCl_3$); IR (neat): 3031, 2932, 2145, 1233, 1114, 1086, 769 cm^{-1}; ^{1}H NMR (500 MHz, $CDCl_3$): δ 7.43-7.06 (m, 25 H, Ar-H), 5.51 (br s, 1 H, PhCH), 5.43 (br s, 1 H, H-1_B), 4.83-4.81 (m, 2 H, 2 PhCH), 4.71 (d, J = 12.0 Hz, 1 H, PhCH), 4.60 (J = 12.0 Hz, 1 H, PhCH), 4.58-4.52 (m, 2 H, 2 PhCH), 4.50 (d, J = 7.5 Hz, 1 H, H-1_A), 4.43 (d, J = 10.5 Hz, 1 H, PhCH), 4.32-4.31 (m, 1 H, H-6_{aA}), 4.26 (d, J = 12.0 Hz, 1 H, PhCH), 4.10 (br s, 1 H, H-2_B), 4.06-4.00 (m, 3 H, H-5_A, H-5_B, H6_{bA}), 3.90 (t, J = 8.5 Hz each, 1 H, H-4_B), 3.86 (dd, J = 8.5 Hz, 3.0 Hz, 1 H, H-3_B), 3.74 (m, 2 H, OCH_2), 3.63 (t, J = 9.0 Hz each, 1 H, H-4_A), 3.48-3.32 (m, 6 H, H-2_A, H-3_A, H-6_{abB}, NCH_2); ^{13}C NMR (125 MHz, $CDCl_3$): δ 137.6-124.9 (Ar-C), 104.1 (C-1_A), 101.3 (PhCH), 99.1 (C-1_B), 81.8 (C-4_A), 80.3 (C-3_A), 79.9 (C-4_B), 77.2 (PhCH$_2$), 75.5 (PhCH$_2$), 75.1 (C-3_B), 74.9 (C-5_B), 73.9 (PhCH$_2$), 73.2 (PhCH$_2$), 71.7 (C-5_A), 70.6 (C-6_A, C-6_B), 68.1 (OCH$_2$), 68.0 (C-2_B), 65.6 (C-2_A), 50.9 (NCH$_2$); ESI MS: 882.3 [M+Na]$^+$; Anal. Calcd. for $C_{49}H_{53}N_3O_{11}$ (859.95): C, 68.44; H, 6.21; found: C, 68.30; H, 6.40.

Allyl 6-*O*-benzoyl-2,3-di-*O*-benzyl-β-D-glucopyranoside (15): To a cooled solution of compound **14** (2 g; 5.0 mmol) in pyridine (20 mL) at 0 °C was added benzoyl chloride (640 µL; 5.5 mmol) and the reaction mixture was stirred at same temperature for 3 h. The solvents were removed under reduced pressure and co-evaporated with toluene to give the crude

product, which was purified over SiO_2 (50% EtOAc/hexane) to give pure compound **15** (1.9 g, 75%) as white foam. $[\alpha]_D^{25}$ –47 (*c* 1.0, $CHCl_3$); IR (neat): 3320, 3130, 1713, 1240, 1116, 696 cm^{-1}; ^{1}H NMR (500 MHz, $CDCl_3$): δ 8.09-7.24 (m, 15 H, Ar-H), 5.92 (m, 1 H, C*H*=CH_2), 5.32-5.17 (m, 2 H, CH=C*H*$_2$), 4.72-4.69 (m, 2 H, 2 PhC*H*), 4.60 (d, *J* = 7.5 Hz, 1 H, H-1), 4.58-4.57 (m, 1 H, H-6_a), 4.55-4.37 (m, 2 H, OC*H*, H-6_b), 4.14-4.09 (m, 1 H, OC*H*), 3.51-3.44 (m, 4 H, H-2, H-3, H-4, H-5); ^{13}C NMR (125 MHz, $CDCl_3$): δ 160.4 (*C*OPh), 138.3-127.5 (Ar-C), 117.3 (*C*H_2), 102.7 (C-1), 83.7 (C-5), 81.7 (C-4), 75.4 (Ph*C*H_2), 74.7 (Ph*C*H_2), 73.7 (C-3), 70.2 (O*C*H_2), 70.0 (C-2), 63.2 (C-6); ESI-MS: 527.2 $[M+Na]^+$; Anal. Calcd. for $C_{30}H_{32}O_7$ (504.57): C, 71.41; H, 6.39; found: C, 71.25; H, 6.55.

Allyl (2,3-di-*O*-benzyl-4,6-*O*-benzylidene-β-D-mannopyranosyl)-(1→4)-6-*O*-benzoyl-2,3-di-*O*-benzyl-β-D-glucopyranoside (16): In a similar manner to a procedure reported earlier [2], to a solution of compound **5** (1 g; 2.03 mmol) in dry CH_2Cl_2 (10 mL) were added BSP (510 mg; 2.44 mmol), TTBP (800 mg; 3.04 mmol) and MS 4 Å (1 g) and the reaction mixture was cooled to −60 °C under argon. To the cooled reaction mixture was added Tf_2O (360 µL; 2.14 mmol) and the reaction mixture was stirred at same temperature for 1 h. After complete consumption of the starting material the reaction mixture was further cooled to −78 °C. To the cold reaction mixture was added compound **15** (1.13 g; 2.23 mmol) and it was stirred for another 2 h at the same temperature. The reaction mixture was warmed to room temperature and diluted with CH_2Cl_2 (50 mL). The organic layer was washed with satd. aq. $NaHCO_3$ (50 mL), H_2O (50 mL), dried (Na_2SO_4) and evaporated to dryness. The crude residue was purified over SiO_2 (10% EtOAc/toluene) to give pure compound **16** (1.23 g, 65%) as colorless oil. $[\alpha]_D^{25}$ +28 (*c* 1.0, $CHCl_3$); IR (neat): 3410, 3019, 1710, 1396, 1047, 696 cm^{-1}; ^{1}H NMR (500 MHz, $CDCl_3$): δ 8.02-7.21 (m, 30 H, Ar-H), 5.92 (m, 1 H, C*H*=CH_2), 5.48 (br s, 1 H, PhC*H*), 5.33-5.18 (m, 2 H, CH=C*H*$_2$), 5.02 (d, *J* = 11.0 Hz, 1 H, PhC*H*), 4.91-4.87 (m, 2 H, 2 PhC*H*), 4.81 (d, *J* = 12.0 Hz, 1 H, PhC*H*), 4.75-4.70 (m, 3 H, 3 PhC*H*), 4.57 (d, *J* = 12.0 Hz, PhC*H*), 4.48-4.46 (m, 3 H, H-1_D, H-1_E, H-6_{aD}), 4.40-4.33 (m, 2 H, H-6_{bD}, OC*H*), 4.16-4.06 (m, 2 H, OC*H*, H-4_E), 3.90 (m, 1 H, H-6_{aE}), 3.87 (m, 2 H, H-2_E, H-3_D), 3.60 (t, *J* = 9.0 Hz each, H-4_D), 3.55-3.44 (m, 4 H, H-2_D, H-3_E, H-5_D, H-6_{bE}), 3.01 (m, 1 H, H-5_E); ^{13}C NMR (125 MHz, $CDCl_3$): δ 166.0 (*C*OPh), 138.9-126.1 (Ar-C), 117.5 (*C*H_2), 102.6 (C-1_D), 101.8 (Ph*C*H), 101.3 (C-1_E), 82.6 (C-4_D), 81.7 (C-4_E, C-5_D), 78.5 (C-3_E), 78.4 (C-3_D), 78.1 (C-2_E), 75.3 (Ph*C*H_2), 75.2 (Ph*C*H_2), 75.0 (Ph*C*H_2), 73.0 (C-2_D), 72.6 (Ph*C*H_2), 70.3 (O*C*H_2), 68.4 (C-6_E), 67.6 (C-5_E), 63.1 (C-6_D); ESI-MS: 957.3 $[M+Na]^+$; Anal. Calcd. for $C_{57}H_{58}O_{12}$ (935.06): C, 73.22; H, 6.25; found: C, 73.05; H, 6.40.

(2,3-Di-*O*-benzyl-4,6-*O*-benzylidene-β-D-mannopyranosyl)-(1→4)-6-*O*-benzoyl-2,3-di-*O*-benzyl-α,β-D-glucopyranosyl trichloroacetimidate (18): To a solution of compound **16** (1 g; 1.07 mmol) in CH_3OH (10 mL) was added $PdCl_2$ (40 mg; 0.21 mmol) at 0 °C and the reaction mixture was stirred at the same temperature for 2 h and 1 h at room temperature. The reaction mixture was filtered through a Celite bed and the filtrate was concentrated under reduced pressure. The crude residue was purified over SiO_2 (50% EtOAc/hexane) to give the hemiacetal derivative **17** (720 mg, 75%) as a pale yellow oil. To a solution of compound **17** (700 mg; 0.78 mmol) in dry CH_2Cl_2 (5 mL) was added CCl_3CN (470 µL; 4.69 mmol) and the mixture was stirred at –10 °C for 15 min. To the cooled solution was added DBU (70 µL; 0.71 mmol) and the reaction mixture was stirred at same temperature for another 30 min. The solvents were removed under reduced pressure at low temperature and the crude residue was purified over SiO_2 (33% EtOAc/hexane) to give the trichloroacetimidate derivative **18** (730 mg, 90%) as colourless oil, which was immediately used for the next step without further characterization.

Phenyl (2,3-di-*O*-benzyl-4,6-*O*-benzylidene-β-D-mannopyranosyl)-(1→4)-(6-*O*-benzoyl-2,3-di-*O*-benzyl-α-D-glucopyranosyl)-(1→3)-2-*O*-benzyl-4,6-*O*-benzylidene-1-thio-α-D-mannopyranoside (19): A solution of compound **18** (695 mg; 0.66 mmol) and compound **6** (250 mg; 0.55 mmol) in dry CH_2Cl_2 (10 mL) was cooled to −10 °C under argon. To the cooled reaction mixture was added TMSOTf (25 µL) and it was stirred at the same temperature for 30 min. The reaction mixture was diluted with CH_2Cl_2 (20 mL) and the organic layer was washed with satd. aq. $NaHCO_3$ (100 mL). The organic layer was dried (Na_2SO_4) and evaporated to dryness. The crude residue was purified over SiO_2 (40% EtOAc/hexane) to give pure compound **19** (330 mg, 45%) as colourless oil. $[\alpha]_D^{25}$ +44 (*c* 1.0, $CHCl_3$); IR (neat): 3400, 3020, 1720, 1390, 1048, 769 cm^{-1}; ^1H NMR (500 MHz, $CDCl_3$): δ 8.05-7.01 (m, 45 H, Ar-H), 5.55 (br s, 1 H, H-1_C), 5.50 (d, *J* = 3.5 Hz, 1 H, H-1_D), 5.46 (br s, 1 H, PhC*H*), 5.45 (br s, 1 H, PhC*H*), 4.91-4.53 (m, 10 H, 10 PhC*H*), 4.52 (br s, 1 H, H-1_E), 4.44-4.31 (m, 5 H, H-3_C, H-2_E, H-3_E, H-6_{abD}), 4.19-4.17 (m, 1 H, H-6_{aC}), 4.12 (br s, 1 H, H-2_C), 4.08 (t, *J* = 8.5 Hz each, 1 H, H-4_E), 4.02 (m, 1 H, H-5_D), 3.95 (t, *J* = 8.0 Hz each, 1 H, H-3_D), 3.92-3.84 (m, 3 H, H-5_C, H-6_{bC}, H-6_{bE}), 3.76 (t, *J* = 9.0 Hz each, 1 H, H-4_D), 3.51-3.44 (m, 3 H, H-2_D, H-4_C, H-6_{bE}), 3.00 (m, 1 H, H-5_E); ^{13}C NMR (125 MHz, $CDCl_3$): δ 166.1 (*C*OPh), 139.1-126.0 (Ar-C), 102.4 (Ph*C*H), 102.1 (C-1_E), 101.3 (Ph*C*H), 96.8 (C-1_D), 87.3 (C-1_C), 80.2 (C-2_C), 79.7 (C-3_C), 79.2 (C-3_D), 78.6 (C-2_D), 78.5 (C-4_E), 78.4 (C-4_D), 78.3 (C-4_C), 77.1 (C-5_C), 75.1 (2 Ph*C*H_2), 74.0 (Ph*C*H_2), 73.2 (C-2_E), 72.5 (Ph*C*H_2), 71.1 (Ph*C*H_2),

69.4 (C-5_D), 68.5 (C-6_E), 68.4 (C-6_D), 67.5 (C-5_E), 65.1 (C-3_E), 63.5 (C-6_D); MALDI-MS: 1349.5 $[M+Na]^+$; Anal. Calcd. for $C_{80}H_{78}O_{16}S$ (1327.53): C, 72.38; H, 5.92; found: C, 72.20; H, 6.10.

2-Azidoethyl (2,3-di-*O*-benzyl-4,6-*O*-benzylidene-β-D-mannopyranosyl)-(1→4)-(6-*O*-benzoyl-2,3-di-*O*-benzyl-α-D-glucopyranosyl)-(1→3)-(2-*O*-benzyl-4,6-*O*-benzylidene-α-D-mannopyranosyl)-(1→2)-(3,4,6-tri-*O*-benzyl-α-D-mannopyranosyl)-(1→3)-2-*O*-benzyl-4,6-*O*-benzylidene-β-D-glucopyranoside (20): To a solution of compound **13** (150 mg; 0.174 mmol) and compound **19** (280 mg; 0.21 mmol) in dry CH_2Cl_2 (5 mL) was added MS 4 Å (200 mg) and the mixture was stirred at room temperature under argon for 30 min and cooled to −10 °C. To the cooled reaction mixture was added NIS (55 mg; 0.23 mmol) followed by TMSOTf (8 µL) and it was stirred at the same temperature for 30 min. The reaction mixture was filtered and washed with CH_2Cl_2 (25 mL) and the combined organic layer was washed with 5% aq. $Na_2S_2O_3$ (25 mL), satd. aq. $NaHCO_3$ (25 mL). The organic layer was dried (Na_2SO_4) and concentrated under reduced pressure. The crude product was purified over SiO_2 (30% EtOAc/hexane) to give pure compound **20** (145 mg, 40%) as a colourless oil. $[\alpha]_D^{25}$ +50 (*c* 1.0, $CHCl_3$); IR (neat): 3400, 3028, 2150, 1720, 1518, 1210, 1047, 669 cm^{-1}; ^1H NMR (500 MHz, $CDCl_3$): δ 7.90-7.00 (m, 65 H, Ar-H), 5.50 (s, 1 H, PhC*H*), 5.42 (s, 1 H, PhC*H*), 5.40 (d, *J* = 2.0 Hz, 1 H, H-1_C), 5.35 (s, 1 H, PhC*H*), 5.32 (d, *J* = 3.5 Hz, 1 H, H-1_D), 4.85 (br s, 1 H, H-1_B), 4.78-4.50 (m, 15 H, 15 PhC*H*), 4.49-4.47 (m, 1 H, H-6_{aD}), 4.46 (d, *J* = 7.5 Hz, 1 H, H-1_A), 4.44 (br s, 1 H, H-1_E), 4.40-4.25 (m, 5 H, H-6_{aA}, H-6_{bD}, 3 PhC*H*), 4.22 (t, *J* = 8.0 Hz each, 1 H, H-4_D), 4.10-3.95 (m, 7 H, H-2_B, H-2_C, H-5_A, H-5_B, H-5_D, H-6_{aE}, H-6_{bA}), 3.92-3.85 (m, 7 H, H-3_B, H-3_C, H-3_D, H-3_E, H-4_B, H-5_C, H-6_{bE}), 3.75-3.67 (m, 2 H, OCH_2), 3.65-3.60 (m, 2 H, H-4_A, H-4_C), 3.55-3.30 (m, 11 H, H-2_A, H-2_D, H-2_E, H-3_A, H-4_E, H-6_{abB}, H-6_{abC}, NCH_2), 2.95-2.88 (m, 1 H, H-5_E); ^{13}C NMR (125 MHz, $CDCl_3$): δ 165.8 (*C*OPh), 139.1-125.9 (Ar-C), 104.2 (C-1_A), 102.2 (C-1_E), 101.8 (Ph*C*H), 101.6 (Ph*C*H), 101.2 (Ph*C*H), 100.8 (C-1_B), 98.9 (C-1_C), 97.1 (C-1_D), 81.8 (C-4_C), 80.6 (C-4_E), 79.5 (C-4_D), 79.4 (C-4_B), 79.3 (C-3_A), 79.1 (C-3_B), 78.9 (C-2_E), 78.5 (C-2_C), 78.4 (C-3_C), 77.5 (C-5_B), 77.3 (C-2_D), 77.1 (C-3_D), 76.6 (C-5_A), 75.6 (C-5D), 75.3 (Ph*C*H_2), 75.2 (2 C, 2 Ph*C*H_2), 75.1 (Ph*C*H_2), 73.7 (Ph*C*H_2), 73.5 (C-3_E), 73.1 (Ph*C*H_2), 72.5 (Ph*C*H_2), 72.4 (Ph*C*H_2), 71.8 (C-2_B), 71.0 (Ph*C*H_2), 68.9 (C-4_A), 68.7 (2C, C-6_A, C-6_C), 68.4 (3C, C-6_B, C-6_E, O*C*H_2), 67.5 (C-5_E), 65.8 (C-2_A), 64.6 (C-5_C), 62.8 (C-6_D), 50.9 (N*C*H_2); MALDI-MS: 2098.8 $[M+Na]^+$; Anal. Calcd. for $C_{123}H_{125}N_3O_{27}$ (2077.31): C, 71.12; H, 6.07; found: C, 70.95; H, 6.20.

2-Azidoethyl (2-*O*-acetyl-4,6-*O*-benzylidene-3-*O*-*p*-methoxybenzyl-α-D-mannopyranosyl)-(1→2)-(3,4,6-tri-*O*-benzyl-α-D-mannopyranosyl)-(1→3)-2-*O*-benzyl-4,6-*O*-benzylidene-β-D-glucopyranoside (21): To a solution of compound **13** (300 mg; 0.35 mmol) and compound **7** (220 mg; 0.42 mmol) in dry CH_2Cl_2 (10 mL) was added MS 4 Å (300 mg) and it was cooled to −50 °C under argon. To the cooled reaction mixture was added NIS (105 mg; 0.46 mmol) followed by TMSOTf (10 µL) and it was stirred at the same temperature for 2 h. The reaction mixture was filtered and washed with CH_2Cl_2 (25 mL) and the combined organic layer was washed with 5% aq. $Na_2S_2O_3$ (25 mL), satd. aq. $NaHCO_3$ (25 mL). The organic layer was dried (Na_2SO_4) and concentrated under reduced pressure. The crude was purified on SiO_2 (30% EtOAc/hexane) to give pure compound **21** (310 mg, 70%) as a yellow oil. $[\alpha]_D^{25}$ +55 (*c* 1.0, $CHCl_3$); IR (neat): 3410, 3020, 2146, 1217, 1116, 969 cm^{-1}; ^{1}H NMR (500 MHz, $CDCl_3$): δ 7.43-6.73 (m, 34 H, Ar-H), 5.50 (br s, 1 H, PhC*H*), 5.48 (br s, 1 H, PhC*H*), 5.42 (br s, 1 H, H-2_C), 5.37 (d, *J* = 1.5 Hz, 1 H, H-1_C), 4.81 (br s, 1 H, H-1_B), 4.79-4.66 (m, 2 H, 2 PhC*H*), 4.60 (br s, 2 H, 2 PhC*H*), 4.59 (d, *J* = 11.0 Hz, 1 H, PhC*H*), 4.53 (d, *J* = 11.0 Hz, 1 H, PhC*H*), 4.48 (d, *J* = 7.5 Hz, 1 H, H-1_A), 4.47 (d, *J* = 11.0 Hz, PhC*H*), 4.45 (d, *J* = 11.0 Hz, 1 H, PhC*H*), 4.33 (br s, 1 H, PhC*H*), 4.31 (m, 1 H, H-6_{aA}), 4.29 (d, *J* = 11.0 Hz, 1 H, PhC*H*), 4.02-3.97 (m, 4 H, H-2_B, H-5_A, H-3_B, H-6_{bA}), 3.95-3.88 (m, 5 H, H-3_C, H-4_B, H-4_C, H-5_B, H-5_C), 3.74-3.69 (m, 3 H, H-6_{aB}, OCH_2), 3.63 (br s, 3 H, OCH_3), 3.61 (t, *J* = 9.0 Hz each, 1 H, H-4_A), 3.46-3.41 (m, 4 H, H-6_{aC}, NCH_2, H-6_{bB}), 3.38-3.30 (m, 3 H, H-2_A, H-3_A, H-6_{bC}); ^{13}C NMR (125 MHz, $CDCl_3$): δ 169.3 (*C*OCH_3), 158.7-112.5 (Ar-C), 104.1 (C-1_A), 101.5 (Ph*C*H), 101.4 (Ph*C*H), 99.9 (C-1_B), 98.7 (C-1_C), 81.7 (C-4_A), 80.5 (C-4_B, C-4_C), 78.3 (C-3_C), 76.1 (C-3_A), 75.4 (Ph*C*H_2), 74.8 (C-3_B), 74.6 (Ph*C*H_2), 73.7 (C-2_C), 73.0 (C-5_A), 72.1 (Ph*C*H_2), 72.0 (2 Ph*C*H_2), 71.6 (C-5_B), 70.0 (C-5_C), 69.3 (C-6_A), 68.7 (C-6_B), 68.6 (C-6_C), 68.2 (O*C*H_2), 65.7 (C-2_B), 64.1 (C-2_A), 55.0 (O*C*H_3), 50.9 (N*C*H_2), 19.9 (CO*C*H_3); MALDI-MS: 1294.5 $[M+Na]^+$; Anal. Calcd. for $C_{72}H_{77}N_3O_{18}$ (1272.39): C, 67.96; H, 6.10; found: C, 67.80; H, 6.30.

2-Azidoethyl (2-*O*-benzyl-4,6-*O*-benzylidene-3-*O*-*p*-methoxybenzyl-α-D-mannopyranosyl)-(1→2)-(3,4,6-tri-*O*-benzyl-α-D-mannopyranosyl)-(1→3)-2-*O*-benzyl-4,6-*O*-benzylidene-β-D-glucopyranoside (22): In a similar manner to a procedure reported earlier [3], to a solution of compound **21** (300 mg; 0.23 mmol) in DMF (5 mL) was added NaOH (300 mg) followed by benzyl bromide (30 µL; 0.25 mmol) and TBAB (20 mg) and stirred at room temperature for 3 h. The reaction mixture was diluted with CH_2Cl_2 (20 mL) and washed with aq. NH_4Cl (10 mL). The organic layer was dried (Na_2SO_4) and concentrated

to give the crude product, which was purified over SiO_2 (25% EtOAc/hexane) to give pure compound **22** (280 mg, 90%) as colourless oil. $[\alpha]_D^{25}$ +31 (*c* 1.0, $CHCl_3$); IR (neat): 3020, 2150, 1390, 1210, 1047, 756 cm^{-1}; ^1H NMR (500 MHz, $CDCl_3$): δ 7.44-6.73 (m, 39 H, Ar-H), 5.52 (br s, 1 H, PhC*H*), 5.49 (br s, 1 H, PhC*H*), 5.33 (br s, 1 H, H-1_C), 5.01 (br s, 1 H, H-1_B), 4.80 (d, *J* = 10.5 Hz, 1 H, PhC*H*), 4.75 (d, *J* = 11.0 Hz, 1 H, PhC*H*), 4.68-4.48 (m, 7 H, 7 PhC*H*), 4.46 (d, *J* = 8.0 Hz, 1 H, H-1_A), 4.45-4.42 (m, 1 H, PhC*H*), 4.29-4.25 (m, 3 H, 2 PhC*H*, H-6_{aA}), 4.10-4.08 (m, 1 H, H-4_B), 4.02-3.95 (m, 4 H, H-4_C, H-5_A, H-5_B, H-6_{bA}), 3.86 (dd, *J* = 9.5 Hz, 3.0 Hz, 1 H, H-3_B), 3.84-3.70 (m, 7 H, H-2_B, H-2_C, H-3_C, H-5_C, H-6_{aB}, OCH_2), 3.68 (br s, 3 H, OCH_3), 3.62 (t, *J* = 9.0 Hz each, 1 H, H-4_A), 3.48-3.42 (m, 4 H, H-6_{aC}, H-6_{bB}, NCH_2), 3.38-3.32 (m, 3 H, H-2_A, H-3_A, H-6_{bC}); ^{13}C NMR (125 MHz, $CDCl_3$): δ 157.8-124.8 (Ar-C), 103.0 (C-1_A), 100.4 (Ph*C*H), 100.1 (Ph*C*H), 99.4 (C-1_B), 97.8 (C-1_C), 81.7 (C-4_A), 80.3 (C-4_B), 79.2 (C-4_C), 79.1 (C-3_C), 76.9 (C-3_A), 75.9 (C-3_B), 75.5 (C-2_C), 75.3 (Ph*C*H_2), 75.0 (C-5_A), 74.9 (Ph*C*H_2), 73.0 (2 Ph*C*H_2), 72.8 (Ph*C*H_2), 72.3 (Ph*C*H_2), 71.7 (C-5_C), 68.8 (C-6_C), 68.6 (C-6_B), 68. 5 (O*C*H_2), 68.3 (C-6_A), 65.7 (C-2_B), 64.5 (C-2_A), 55.0 (O*C*H_3), 50.9 (N*C*H_2); MALDI-MS: 1342.5 $[M+Na]^+$; Anal. Calcd. for $C_{77}H_{81}N_3O_{17}$ (1320.47): C, 70.04; H, 6.18; found: C, 69.88; H, 6.34.

2-Azidoethyl (2-*O*-benzyl-4,6-*O*-benzylidene-α-D-mannopyranosyl)-(1→2)-(3,4,6-tri-*O*-benzyl-α-D-mannopyranosyl)-(1→3)-2-*O*-benzyl-4,6-*O*-benzylidene-β-D-glucopyranoside (23): In a similar manner to a procedure reported earlier [3], to a solution of compound **22** (250 mg; 0.19 mmol) in CH_2Cl_2-H_2O (6 mL; 5:1 v/v) was added DDQ (50 mg; 0.23 mmol) and the reaction mixture was stirred at room temperature for 3 h. The reaction mixture was diluted with CH_2Cl_2 (20 mL) and washed with H_2O (20 mL). The organic layer was dried (Na_2SO_4) and concentrated. The crude product was purified over SiO_2 (50% EtOAc/hexane) to give pure compound **23** (190 mg, 84%) as yellow oil. $[\alpha]_D^{25}$ – 10 (*c* 1.0, $CHCl_3$); IR (neat): 3430, 3029, 2155, 1517, 1210, 976 cm^{-1}; ^1H NMR (500 MHz, $CDCl_3$): δ 7.42-7.09 (m, 35 H, Ar-H), 5.50 (br s, 1 H, PhC*H*), 5.44 (br s, 1 H, PhC*H*), 5.34 (br s, 1 H, H-1_C), 5.11 (br s, 1 H, H-1_B), 4.83-4.79 (m, 2 H, 2 PhC*H*), 4.68 (d, *J* = 11.0 Hz, 1 H, PhC*H*), 4.61-4.59 (m, 2 H, 2 PhC*H*), 4.49 (d, *J* = 7.5 Hz, 1 H, H-1_A), 4.47 (d, *J* = 11.0 Hz, 1 H, PhC*H*), 4.37-4.28 (m, 4 H, 3 PhC*H*, H-6_{aA}), 4.04-3.98 (m, 5 H, H-4_B, H-4_C, H-5_A, H-5_B, H-6_{bA}), 3.93-3.91 (m, 2 H, H-2_B, H-3_B), 3.84-3.71 (m, 5 H, H-2_C, H-3_C, H-5_C, OCH_2), 6.63-3.58 (m, 2 H, H-4_A, H-6_{aC}), 3.51-3.40 (m, 7 H, H-2_A, H-3_A, H-6_{abB}, H-6_{bC}, NCH_2); ^{13}C NMR (125 MHz, $CDCl_3$): δ 137.6-124.9 (Ar-C), 104.1 (C-1_A), 101.9 (Ph*C*H), 101.6 (Ph*C*H), 99.6 (C-1_B), 98.9 (C-1_C), 81.8 (C-4_A), 80.5 (C-4_B), 79.4 (C-4_C), 78.5 (C-3_C), 77.1 (C-3_B), 75.7 (C-3_A), 75.4 (Ph*C*H_2), 75.3 (C-

2_C), 75.1 (PhCH_2), 74.8 (C-5_A), 73.0 (PhCH_2), 72.9 (PhCH_2), 72.7 (PhCH_2), 71.8 (C-5_B), 68.6 (C-6_A), 68.4 (C-6_B), 68.3 (C-6_C), 68.2 (OCH_2), 68.1 (C-5_C), 65.7 (C-2_B), 63.8 (C-2_A), 50.9 (NCH_2); MALDI-MS: 1222.4 $[M+Na]^+$; Anal. Calcd. for $C_{69}H_{73}N_3O_{16}$ (1200.32): C, 69.04; H, 6.13; found: C, 68.86; H, 6.30.

2-Azidoethyl (2,3-di-*O*-benzyl-4,6-*O*-benzylidene-β-D-mannopyranosyl)-(1→4)-(6-*O*-benzoyl-2,3-di-*O*-benzyl-α-D-glucopyranosyl)-(1→3)-(2-*O*-benzyl-4,6-*O*-benzylidene-α-D-mannopyranosyl)-(1→2)-(3,4,6-tri-*O*-benzyl-α-D-mannopyranosyl)-(1→3)-2-*O*-benzyl-4,6-*O*-benzylidene-β-D-glucopyranoside (20): A solution of compound **23** (150 mg; 0.12 mmol) and compound **18** (155 mg; 0.15 mmol) in dry CH_2Cl_2 (5 mL) was cooled to −10 °C. To the cooled reaction mixture was added TMSOTf (15 µL) and it was stirred at the same temperature for 30 min. The reaction mixture was diluted with CH_2Cl_2 (25 mL) and the organic layer was successively washed with satd. aq. $NaHCO_3$ (20 mL) and H_2O (20 mL). The organic layer was dried (Na_2SO_4) and concentrated to give the crude product, which was purified over SiO_2 (40% EtOAc/hexane) to give pure compound **20** (185 mg, 70%) as colourless oil. Analytical data obtained was identical to the mentioned earlier.

2-Aminoethyl (β-D-mannopyranosyl)-(1→4)-(α-D-glucopyranosyluronic acid)-(1→3)-(α-D-mannopyranosyl)-(1→2)-(α-D-mannopyranosyl)-(1→3)-β-D-glucopyranoside (1): A solution of compound **20** (150 mg, 0.072 mmol) in 0.1 M CH_3ONa in CH_3OH (10 mL) was stirred at room temperature for 4 h and neutralized with Dowex 50W X8 (H^+) resin. The reaction mixture was filtered and concentrated under reduced pressure. To a solution of the crude product in CH_2Cl_2 (20 mL) and H_2O (3 mL) were successively added aq. 1 M NaBr (1 mL), aq. 1 M TBAB (2 mL), TEMPO (75 mg, 0.48 mmol), satd. $NaHCO_3$ (8 mL) and 4% aq. NaOCl (10 mL) and the reaction mixture was allowed to stir at 5 °C for 3 h and neutralized with 1 M aq. HCl. To the reaction mixture were added *tert*-butanol (5 mL), 2-methyl-but-2-ene (5 mL; 2 M solution in THF), aq. $NaClO_2$ (6 mL, 20%), and aq. NaH_2PO_4 (6 mL, 20%) and it was stirred at room temperature for 3 h. The reaction mixture was diluted with satd. aq. NaH_2PO_4 and extracted with CH_2Cl_2 (30 mL). The organic layer was washed with water, dried (Na_2SO_4), and concentrated to dryness. To a solution of the crude product in CH_3OH (15 mL) was added 20% $Pd(OH)_2/C$ (100 mg) and the reaction mixture was allowed to stir at room temperature under hydrogen for 24 h. The reaction mixture was filtered through a Celite bed and washed with CH_3OH-H_2O (60 mL; 5:1 v/v). The combined filtrate was evaporated under reduced pressure to furnish compound **1**, which was purified through a

Sephadex LH-20 column using CH_3OH-H_2O (6:1) as eluant to give pure compound **1** (40 mg; 61%); white powder; $[\alpha]_D^{25}$ +21 (*c* 1.0, CH_3OH); IR (KBr): 3020, 2362, 1754, 1721, 1216, 929, 760 cm^{-1}; ^{1}H NMR (500 MHz, D_2O): δ 5.47 (br s, 1 H, H-1_B), 5.27 (d, *J* = 3.5 Hz, 1 H, H-1_D), 5.05 (br s, 1 H, H-1_C), 4.68 (br s, 1 H, H-1_E), 4.53 (d, *J* = 7.5 Hz, 1 H, H-1_A), 4.26-4.24 (m, 1 H, H-2_C), 4.20-4.06 (m, 3 H, H-2_B, H-2_E, H-5_D), 4.00-3.90 (m, 8 H, H-3_C, H-3_D, H-3_E, H-4_E, H-6_{aA}, H-6_{aB}, H-6_{aE}, OC*H*), 3.88-3.72 (m, 9 H, H-3_B, H-4_C, H-5_B, H-6_{bA}, H-6_{bB}, H-6_{abC}, H-6_{bE}, OC*H*), 3.70-3.65 (m, 3 H, H-3_A, H-4_B, H-5_C), 3.62-3.57 (m, 3 H, H-2_D, H-4_A, H-4_D), 3.54-3.50 (m, 1 H, H-5_A), 3.48-3.33 (m, 2 H, H-2_A, H-5_E), 3.30-3.27 (m, 2 H, NCH_2);^{13}C NMR (125 MHz, D_2O): δ 175.0 (*C*OOH), 102.2 (C-1_A), 102.1 (C-1_C), 100.2 (C-1_E), 99.9 (C-1_D), 99.4 (C-1_B), 82.1 (C-3_A), 78.7 (C-2_D), 78.6 (2 C, C-3_C, C-4_D), 76.4 (C-2_B), 75.7 (C-5_A), 73.2 (C-5_E), 72.9 (C-3_E), 72.8 (C-5_B), 71.7 (2 C, C-2_A, C-2_E), 71.5 (C-5_C), 71.3 (C-5_D), 70.9 (C-4_A), 70.6 (C-2_C), 70.1 (C-3_B), 69.9 (C-3_D), 69.8 (C-4_C), 66.7 (C-4_B), 65.8 (2 C, C-4_E, O*C*H_2), 60.9 (C-6_E), 60.6 (C-6_C), 60.4 (C-6_B), 60.2 (C-6_A), 39.4 (N*C*H_2); MALDI-MS: 934.2 $[M+Na]^+$; Anal. Calcd. for $C_{32}H_{53}N_3O_{27}$ (911.76): C, 42.15; H, 5.86; found: C, 42.00; H, 6.05.

Reference

[1] Mandal, P. K.; Misra, A. K. *Glycoconj. J.* **2008**, *25*, 713-722.

[2] Si, A.; Misra, A. K. *Tetrahedron* **2016**, *72*, 4435-4441.

[3] Panchadhayee, R.; Misra, A. K. *Glycoconj. J.* **2008**, *25*, 817-826.

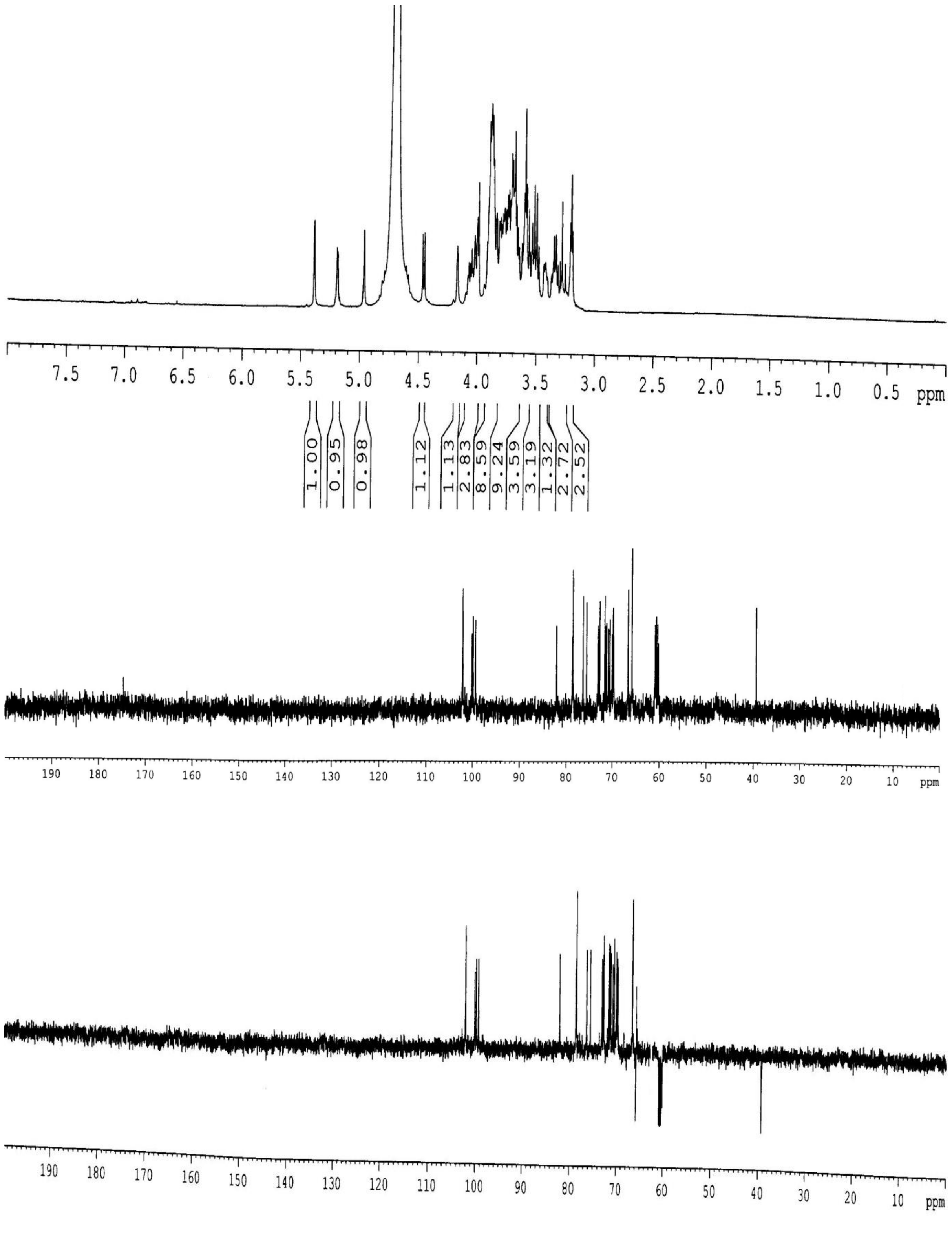

^{1}H and ^{13}C NMR spectra of 2-aminoethyl (β-D-mannopyranosyl)-(1→4)-(α-D-glucopyranosyluronic acid)-(1→3)-(α-D-mannopyranosyl)-(1→2)-(α-D-mannopyranosyl)-(1→3)-β-D-glucopyranoside (**1**) (D_2O).

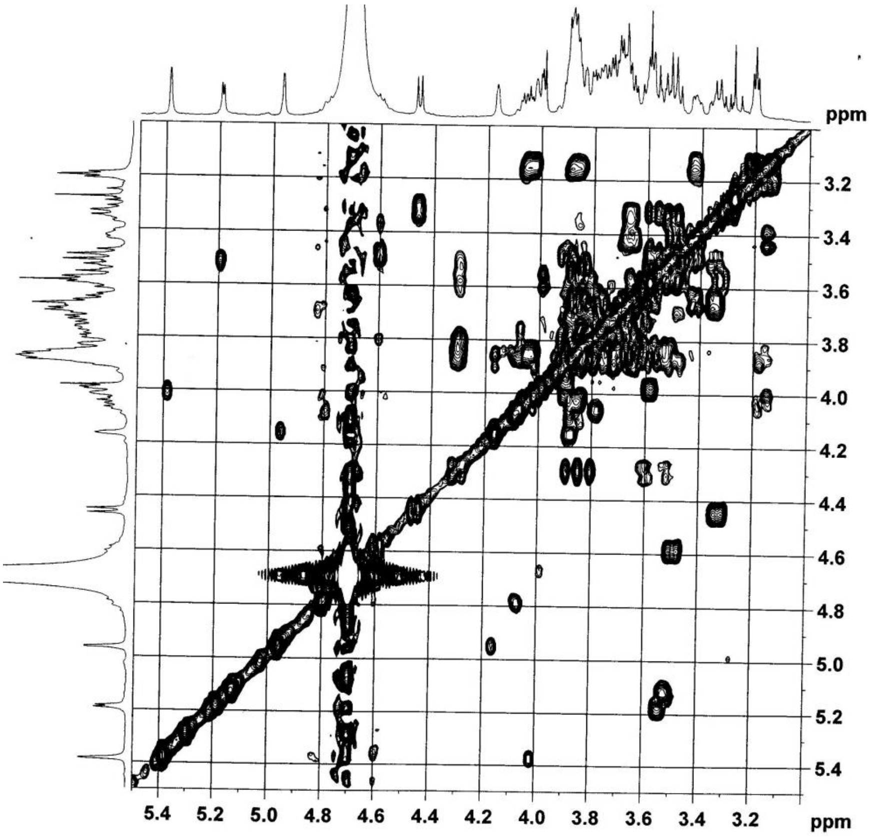

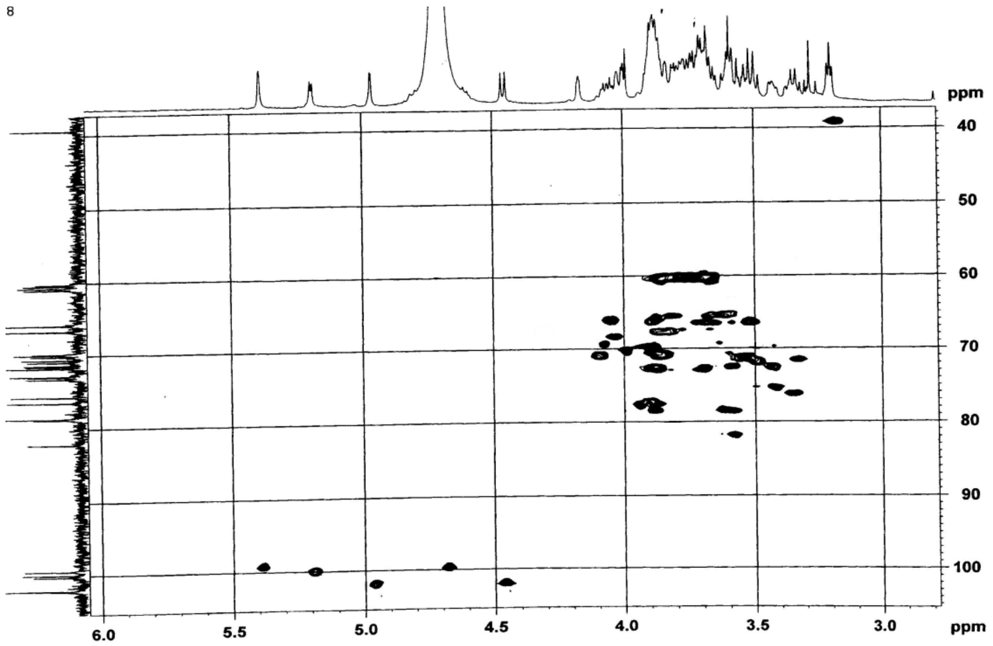

2D COSY and HSQC NMR spectra of 2-aminoethyl (β-D-mannopyranosyl)-(1→4)-(α-D-glucopyranosyluronic acid)-(1→3)-(α-D-mannopyranosyl)-(1→2)-(α-D-mannopyranosyl)-(1→3)-β-D-glucopyranoside (**1**) (D_2O) (selected region).

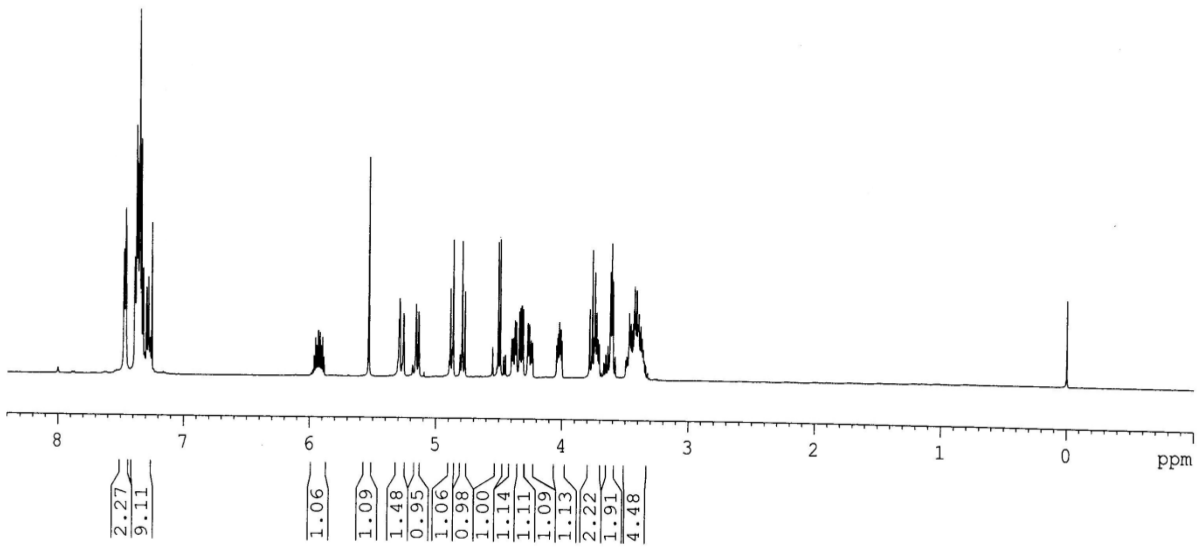

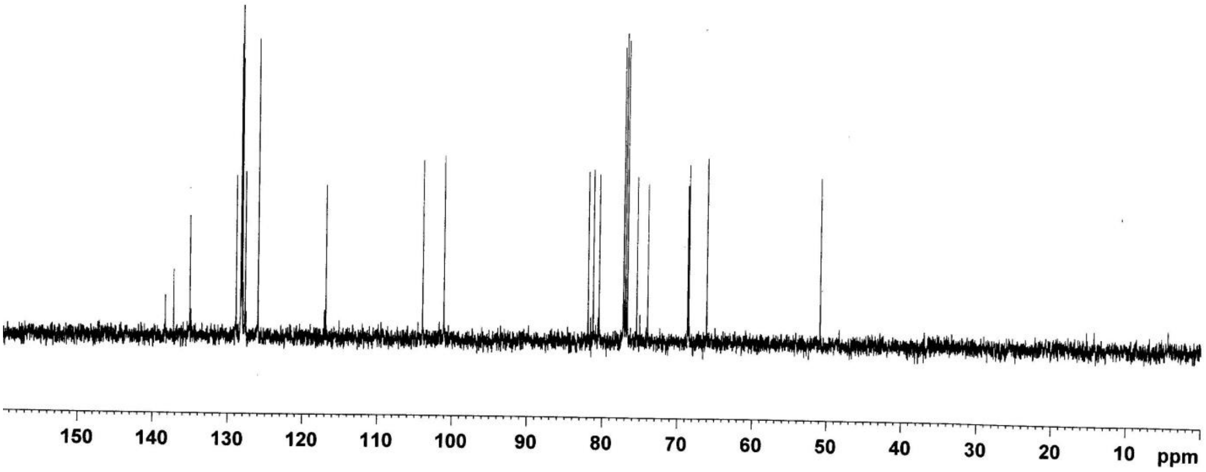

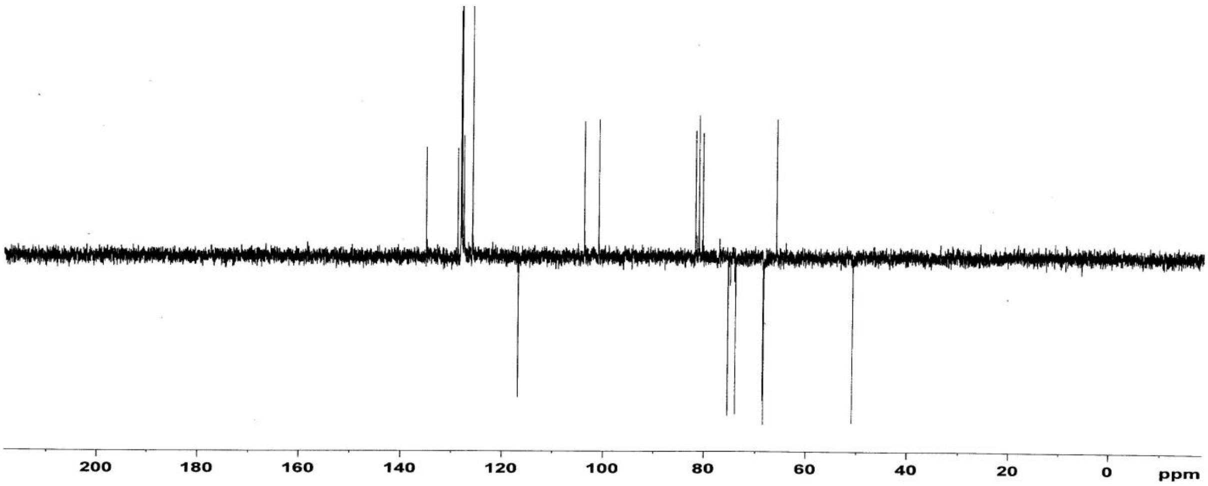

^{1}H and ^{13}C NMR spectra of 2-azidoethyl 3-O-allyl-2-O-benzyl-4,6-O-benzylidene-β-D-glucopyranoside (**10**) ($CDCl_3$).

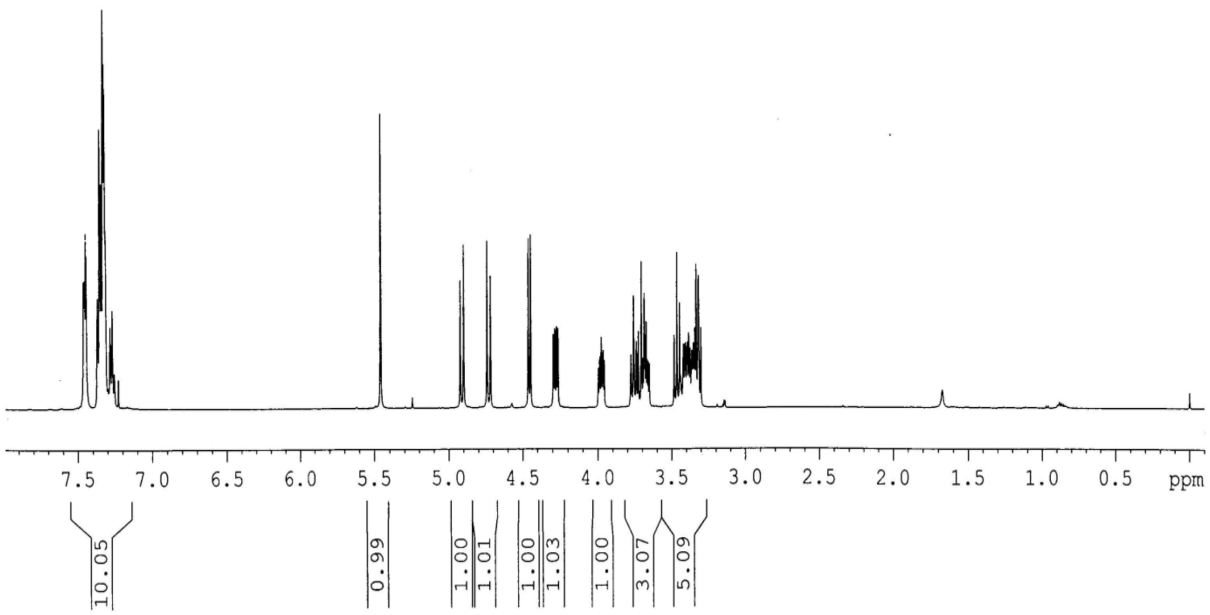

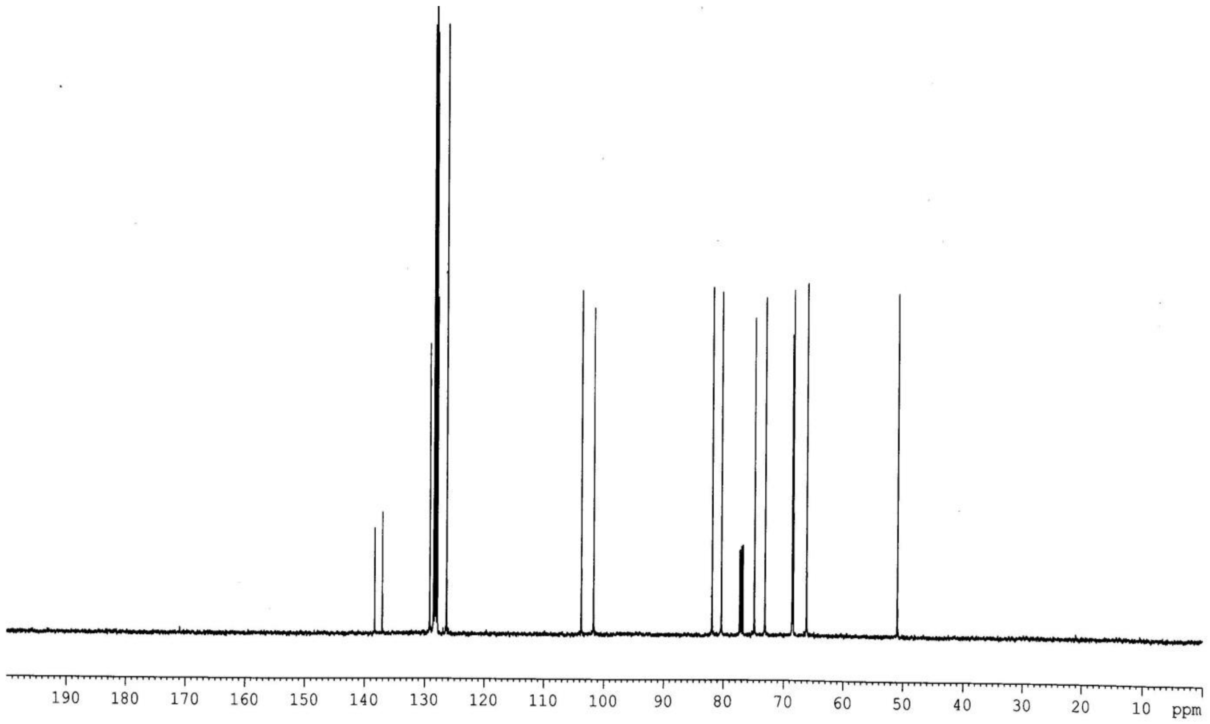

^{1}H and ^{13}C NMR spectra of 2-azidoethyl 2-O-benzyl-4,6-O-benzylidene-β-D-glucopyranoside (**11**) ($CDCl_3$).

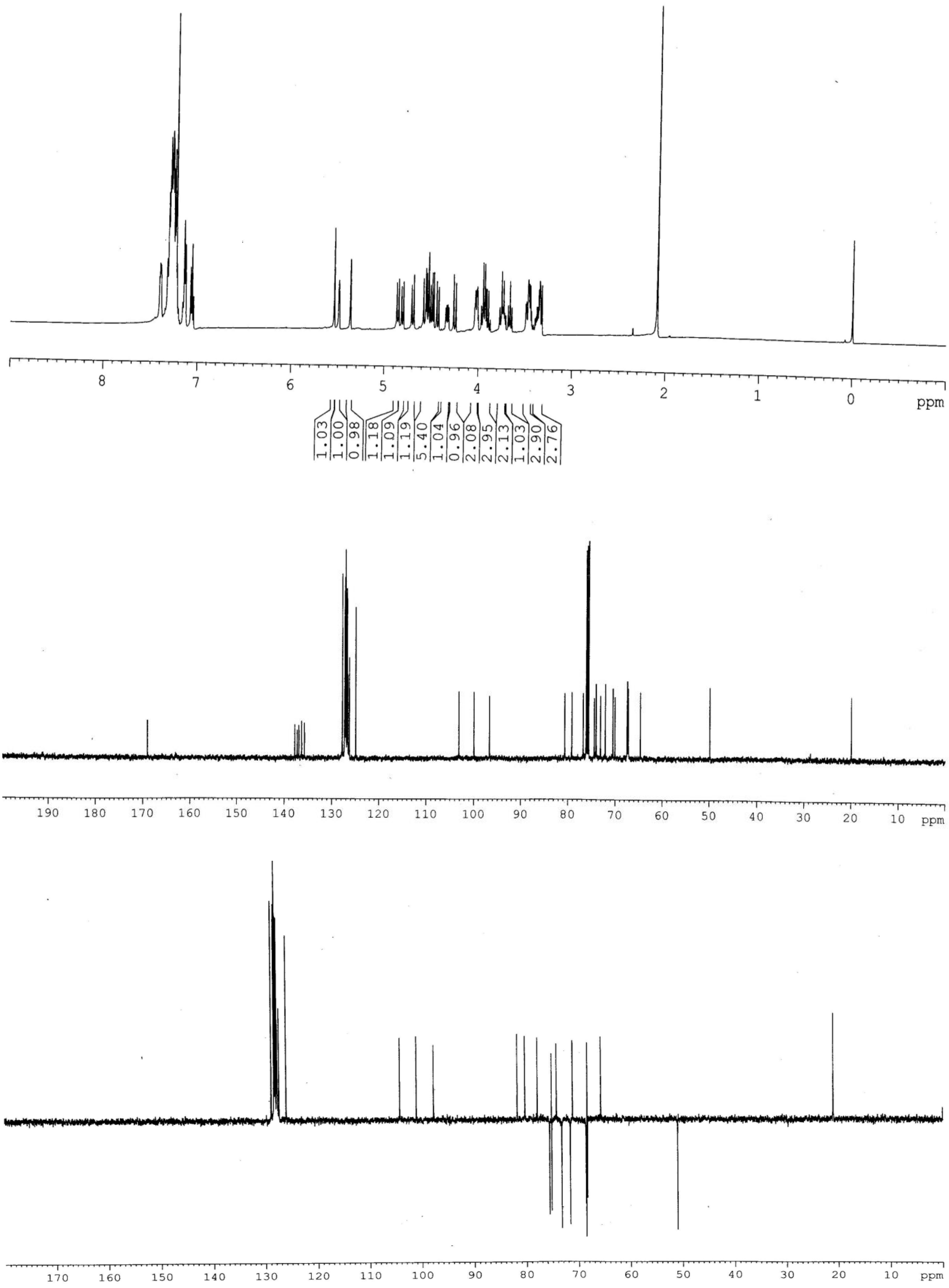

^{1}H and ^{13}C NMR spectra of 2-azidoethyl (2-*O*-acetyl-3,4,6-tri-*O*-benzyl-α-D-mannopyranosyl)-(1→3)-2-*O*-benzyl-4,6-*O*-benzylidene-β-D-glucopyranoside (**12**) ($CDCl_3$).

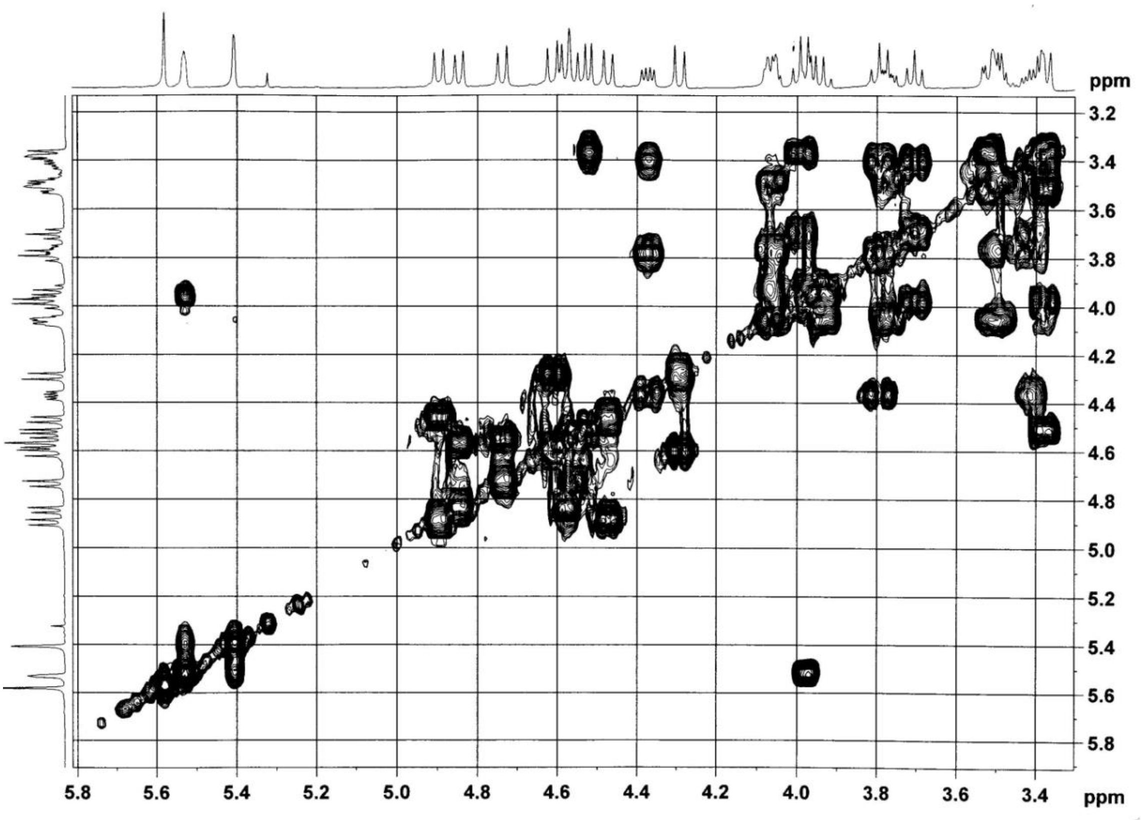

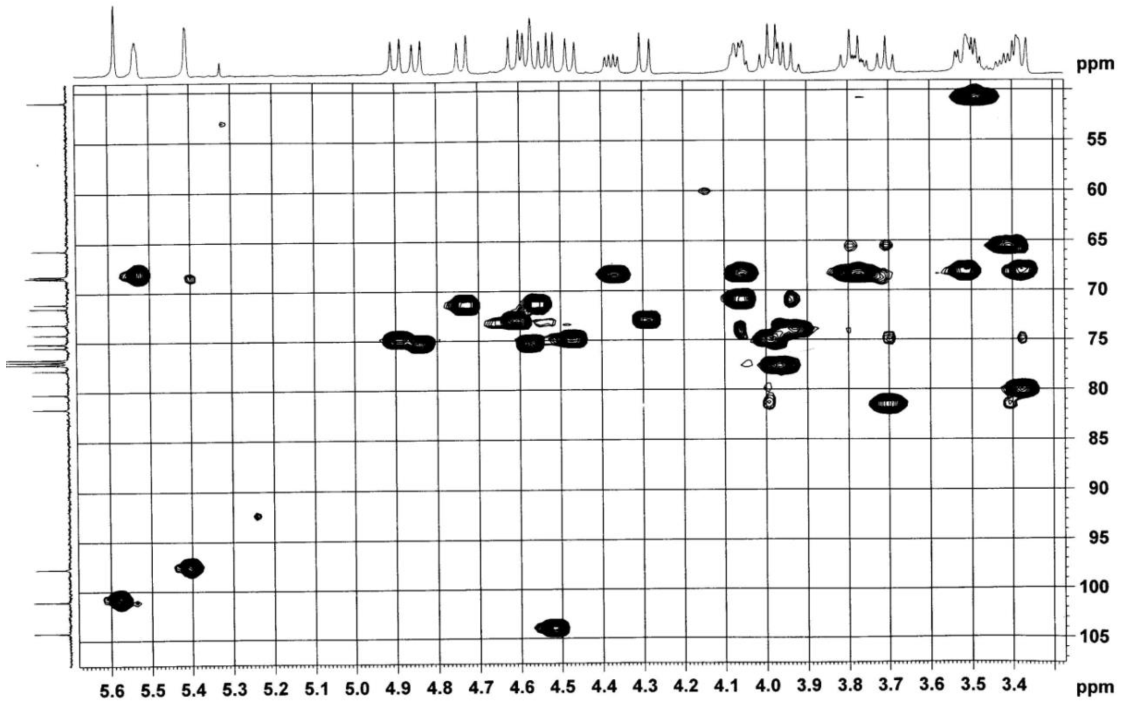

2D COSY and HSQC NMR spectra of 2-azidoethyl (2-*O*-acetyl-3,4,6-tri-*O*-benzyl-α-D-mannopyranosyl)-(1→3)-2-*O*-benzyl-4,6-*O*-benzylidene-β-D-glucopyranoside (**12**) ($CDCl_3$) (selected region).

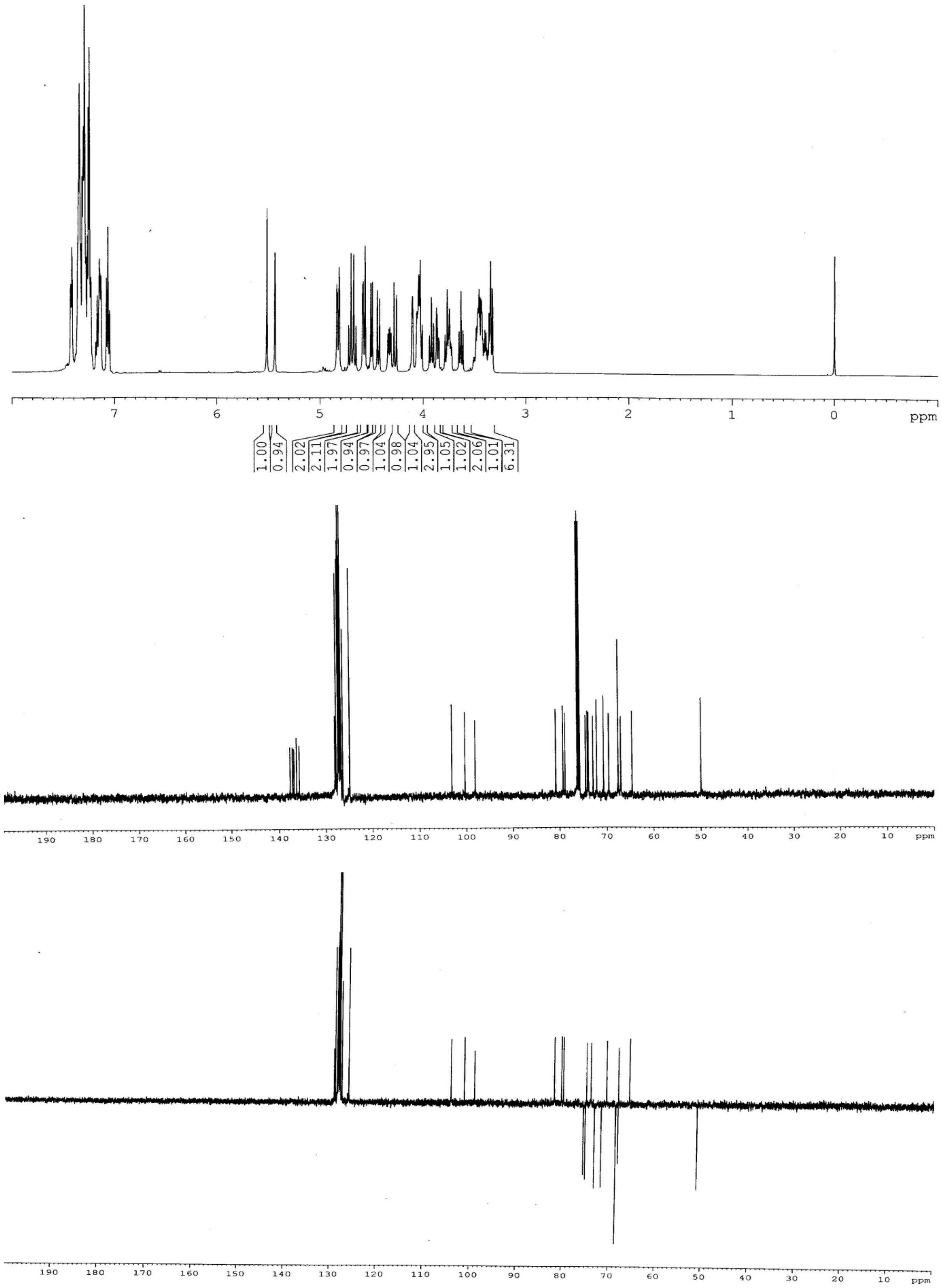

^{1}H and ^{13}C NMR spectra of 2-azidoethyl (3,4,6-tri-*O*-benzyl-α-D-mannopyranosyl)-(1→3)-2-*O*-benzyl-4,6-*O*-benzylidene-β-D-glucopyranoside (**13**) ($CDCl_3$).

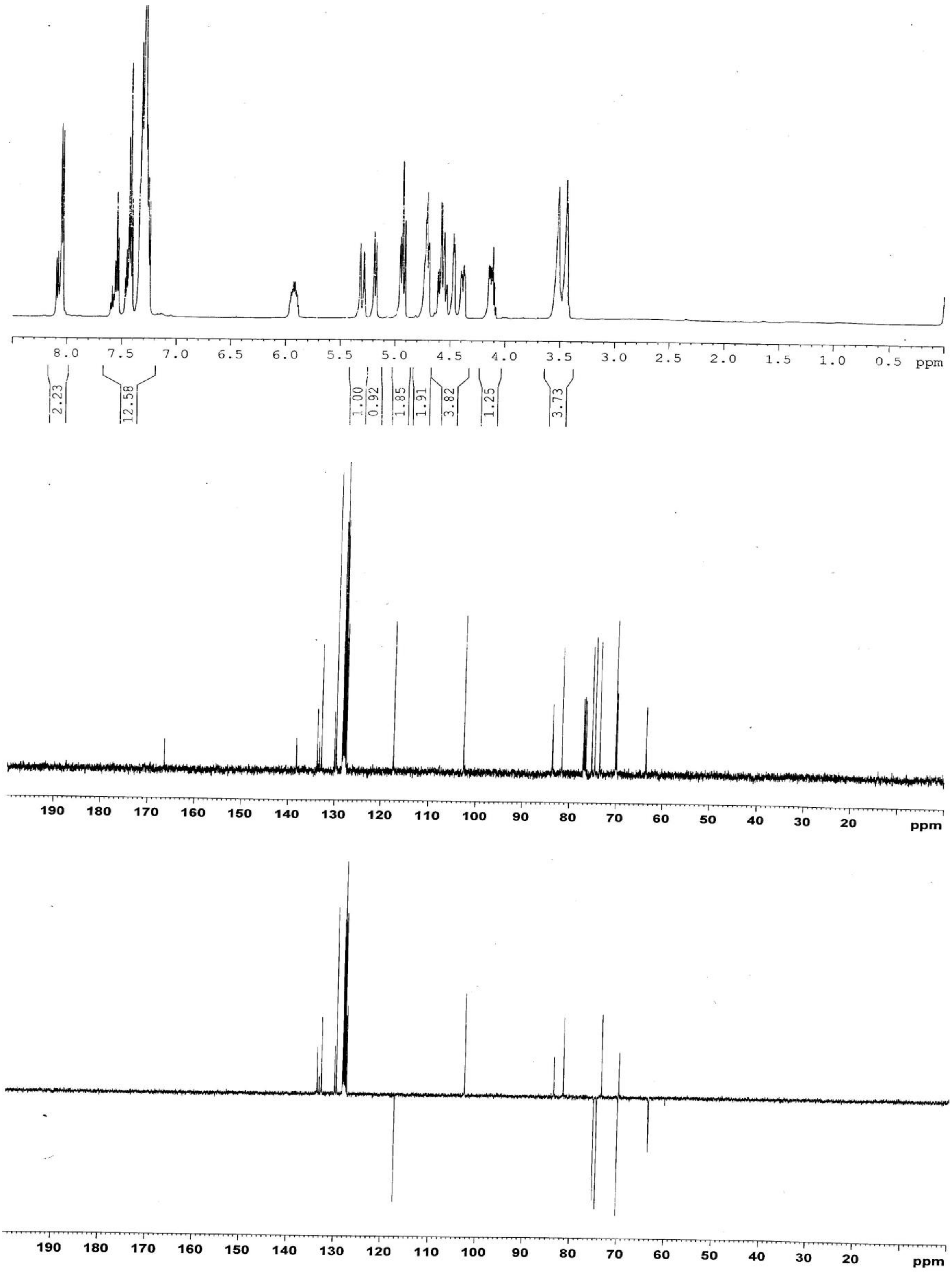

^{1}H and ^{13}C NMR spectra of allyl 6-*O*-benzoyl-2,3-di-*O*-benzyl-β-D-glucopyranoside (**15**) ($CDCl_3$).

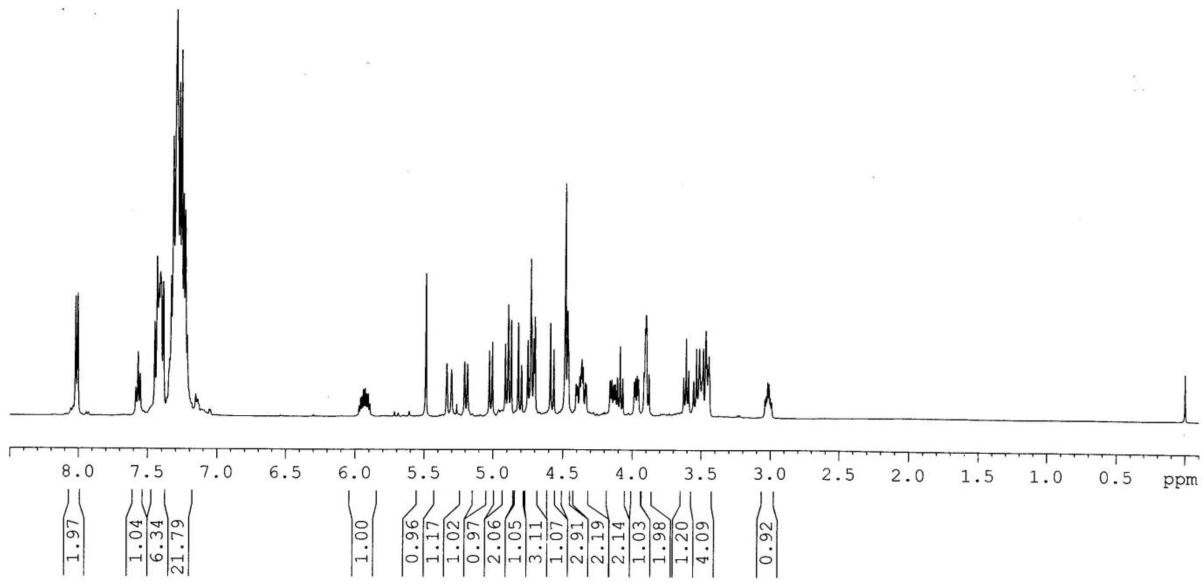

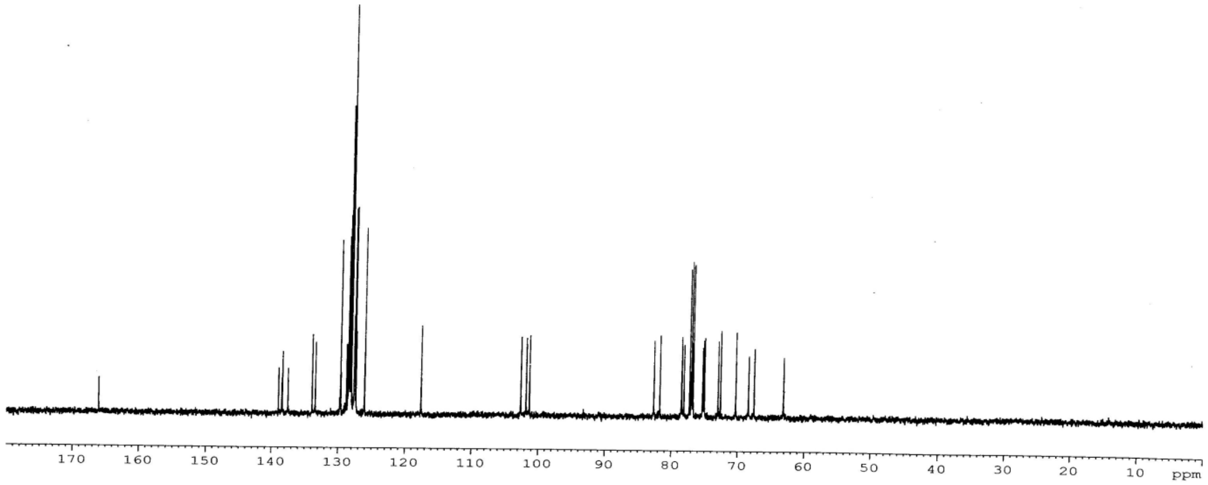

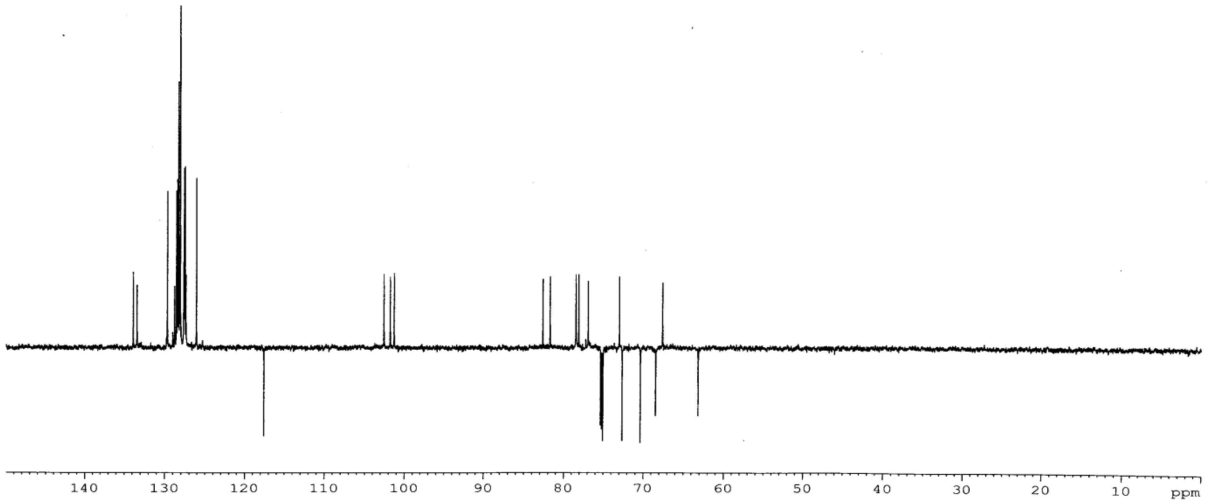

^{1}H and ^{13}C NMR spectra of allyl (2,3-di-*O*-benzyl-4,6-*O*-benzylidene-β-D-mannopyranosyl)-(1→4)-6-*O*-benzoyl-2,3-di-*O*-benzyl-β-D-glucopyranoside (**16**) ($CDCl_3$).

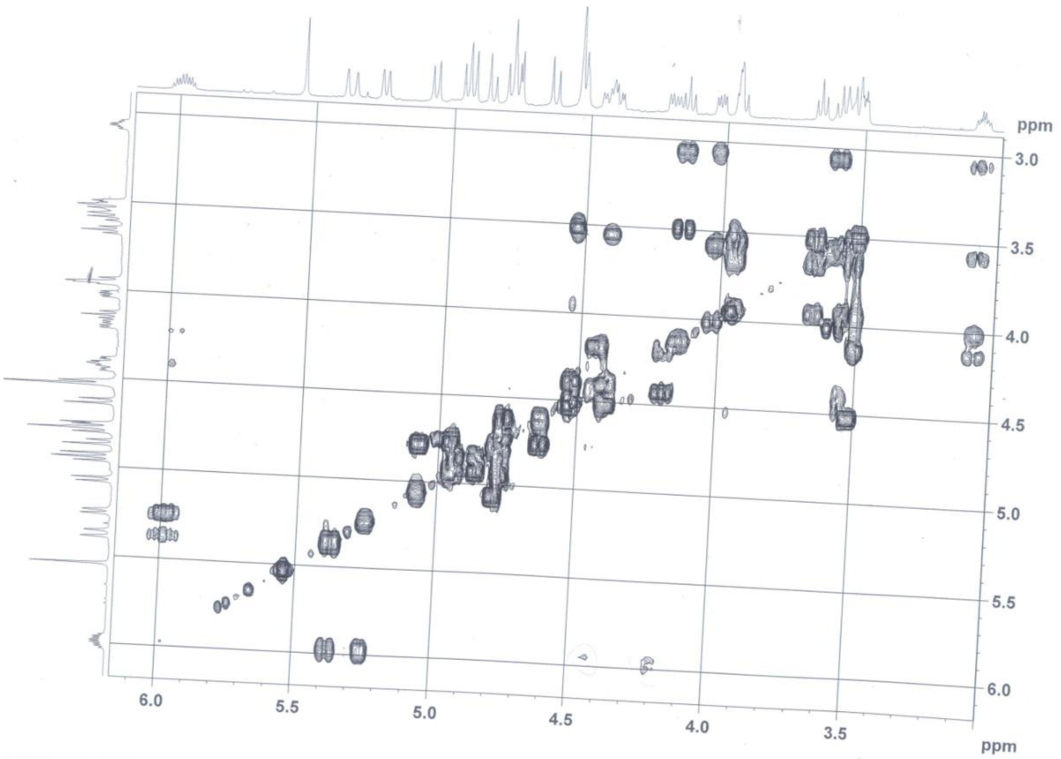

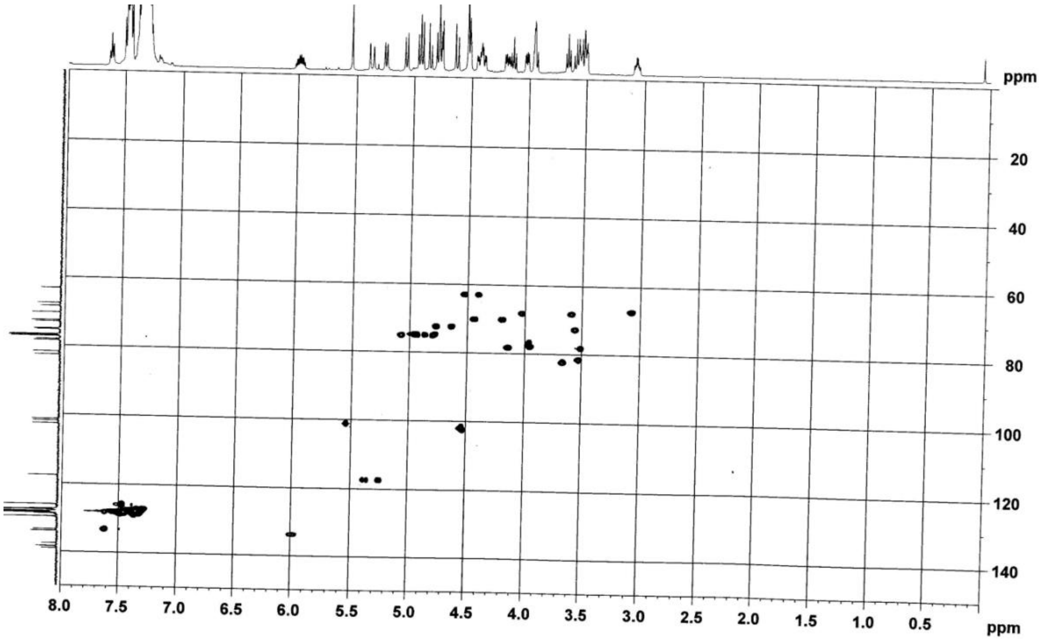

2D COSY and HSQC NMR spectra of allyl (2,3-di-*O*-benzyl-4,6-*O*-benzylidene-β-D-mannopyranosyl)-(1→4)-6-*O*-benzoyl-2,3-di-*O*-benzyl-β-D-glucopyranoside **(16)** ($CDCl_3$) (selected region).

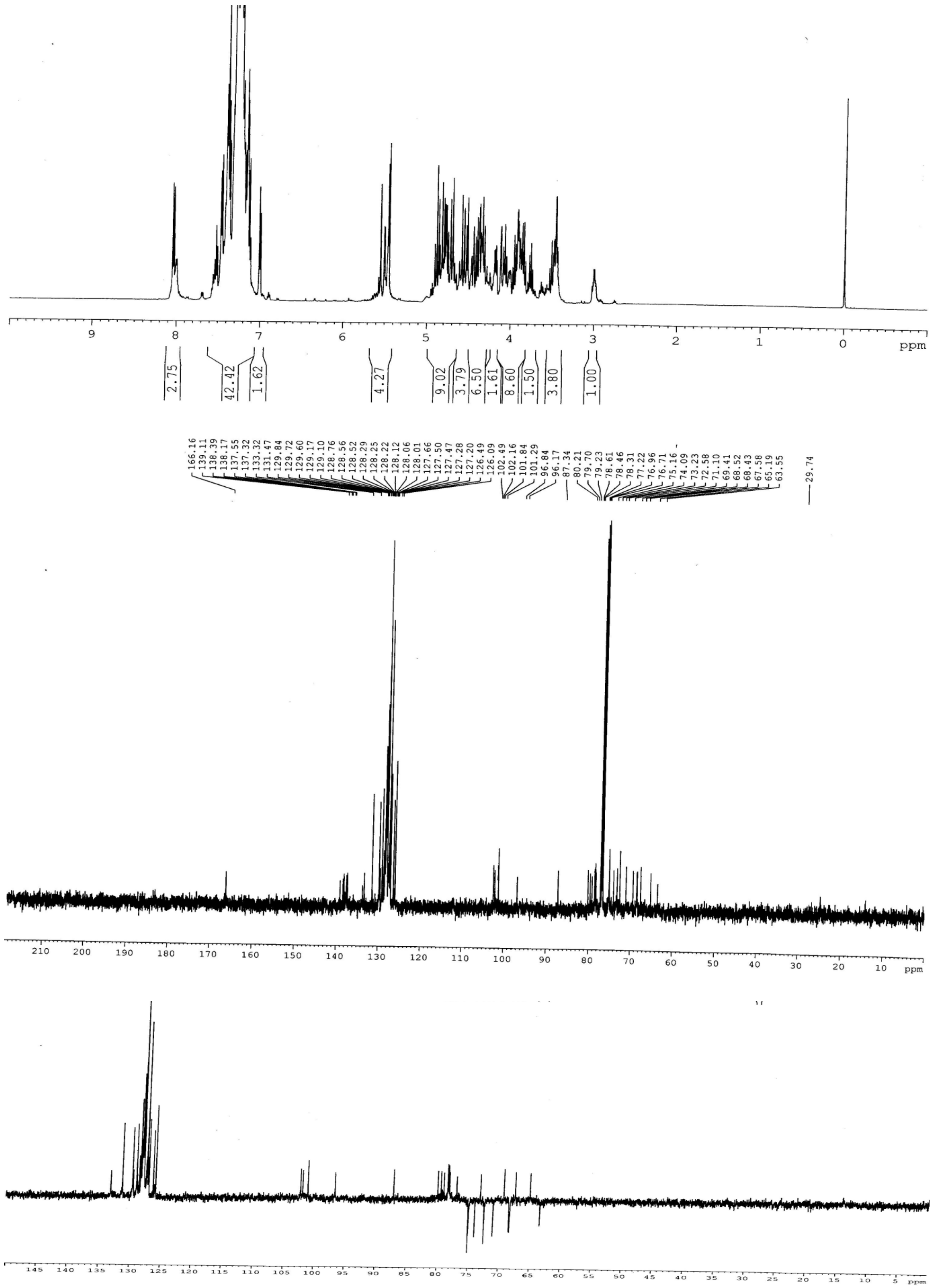

^{1}H and ^{13}C NMR spectra of phenyl (2,3-di-*O*-benzyl-4,6-*O*-benzylidene-β-D-mannopyranosyl)-(1→4)-(6-*O*-benzoyl-2,3-di-*O*-benzyl-α-D-glucopyranosyl)-(1→3)-2-*O*-benzyl-4,6-*O*-benzylidene-1-thio-α-D-mannopyranoside (**19**) ($CDCl_3$).

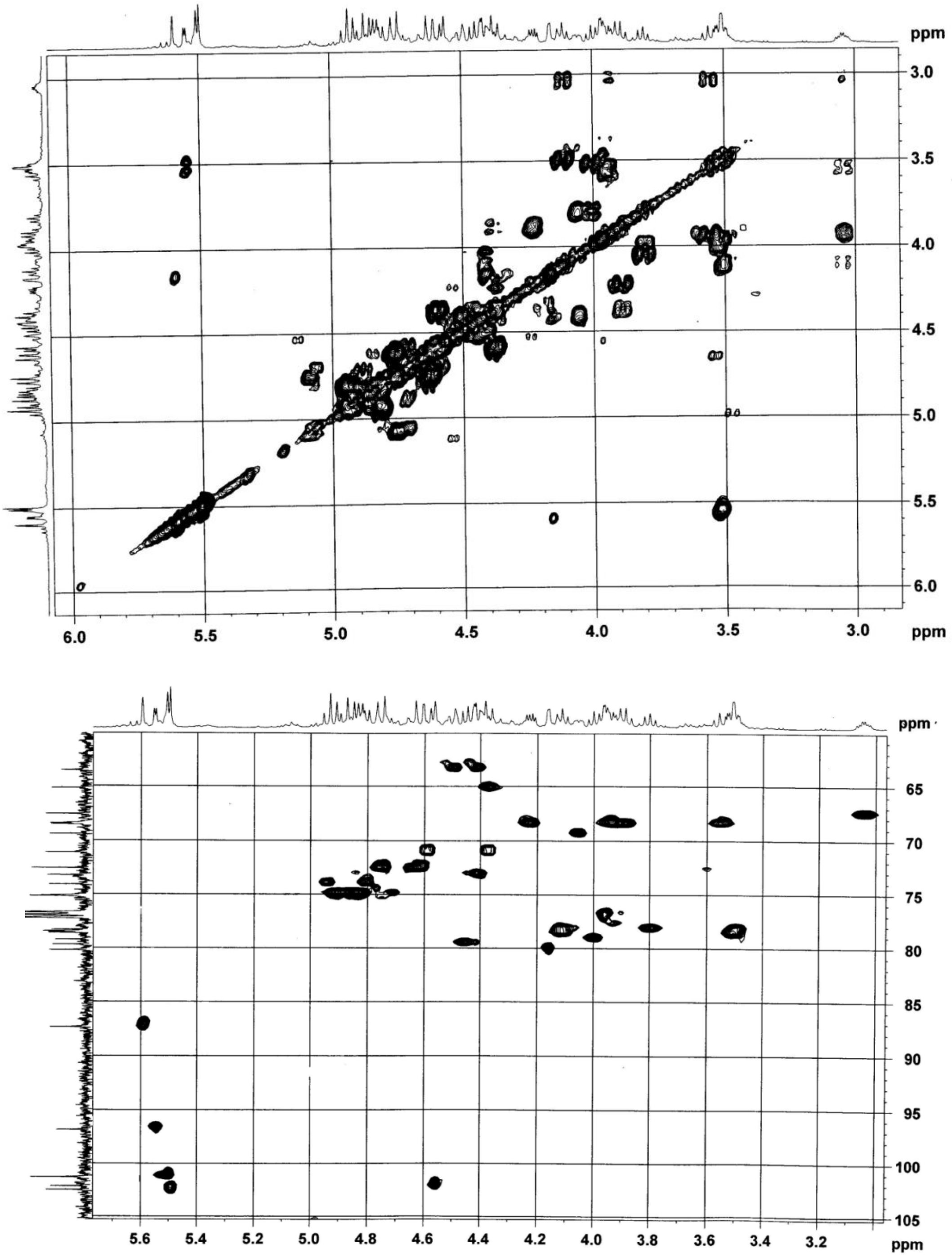

2D COSY and HSQC NMR spectra of phenyl (2,3-di-*O*-benzyl-4,6-*O*-benzylidene-β-D-mannopyranosyl)-(1→4)-(6-*O*-benzoyl-2,3-di-*O*-benzyl-α-D-glucopyranosyl)-(1→3)-2-*O*-benzyl-4,6-*O*-benzylidene-1-thio-α-D-mannopyranoside (**19**) ($CDCl_3$) (selected region).

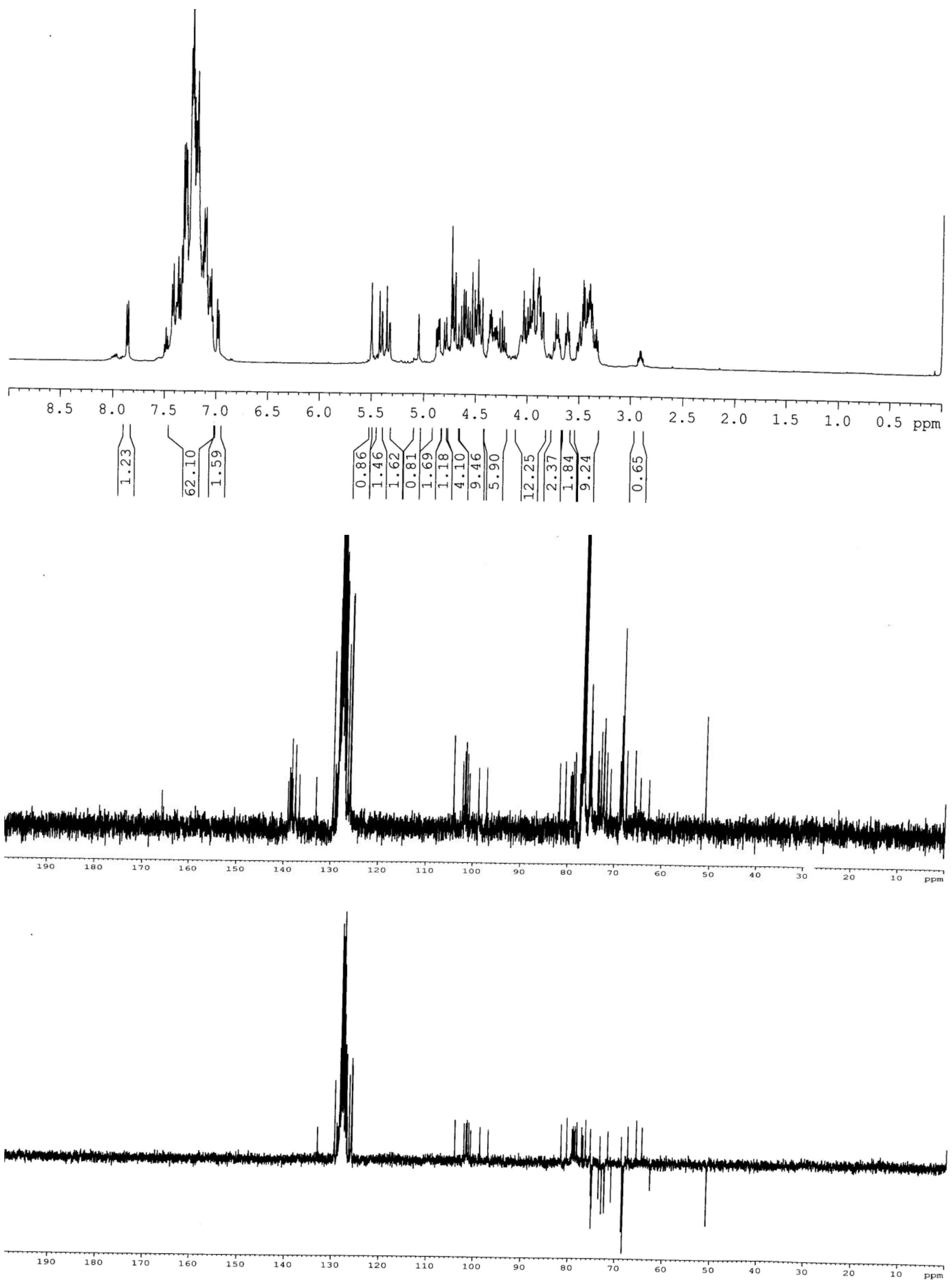

^{1}H and ^{13}C NMR spectra of 2-azidoethyl (2,3-di-*O*-benzyl-4,6-*O*-benzylidene-β-D-mannopyranosyl)-(1→4)-(6-*O*-benzoyl-2,3-di-*O*-benzyl-α-D-glucopyranosyl)-(1→3)-(2-*O*-benzyl-4,6-*O*-benzylidene-α-D-mannopyranosyl)-(1→2)-(3,4,6-tri-*O*-benzyl-α-D-mannopyranosyl)-(1→3)-2-*O*-benzyl-4,6-*O*-benzylidene-β-D-glucopyranoside (**20**) ($CDCl_3$).

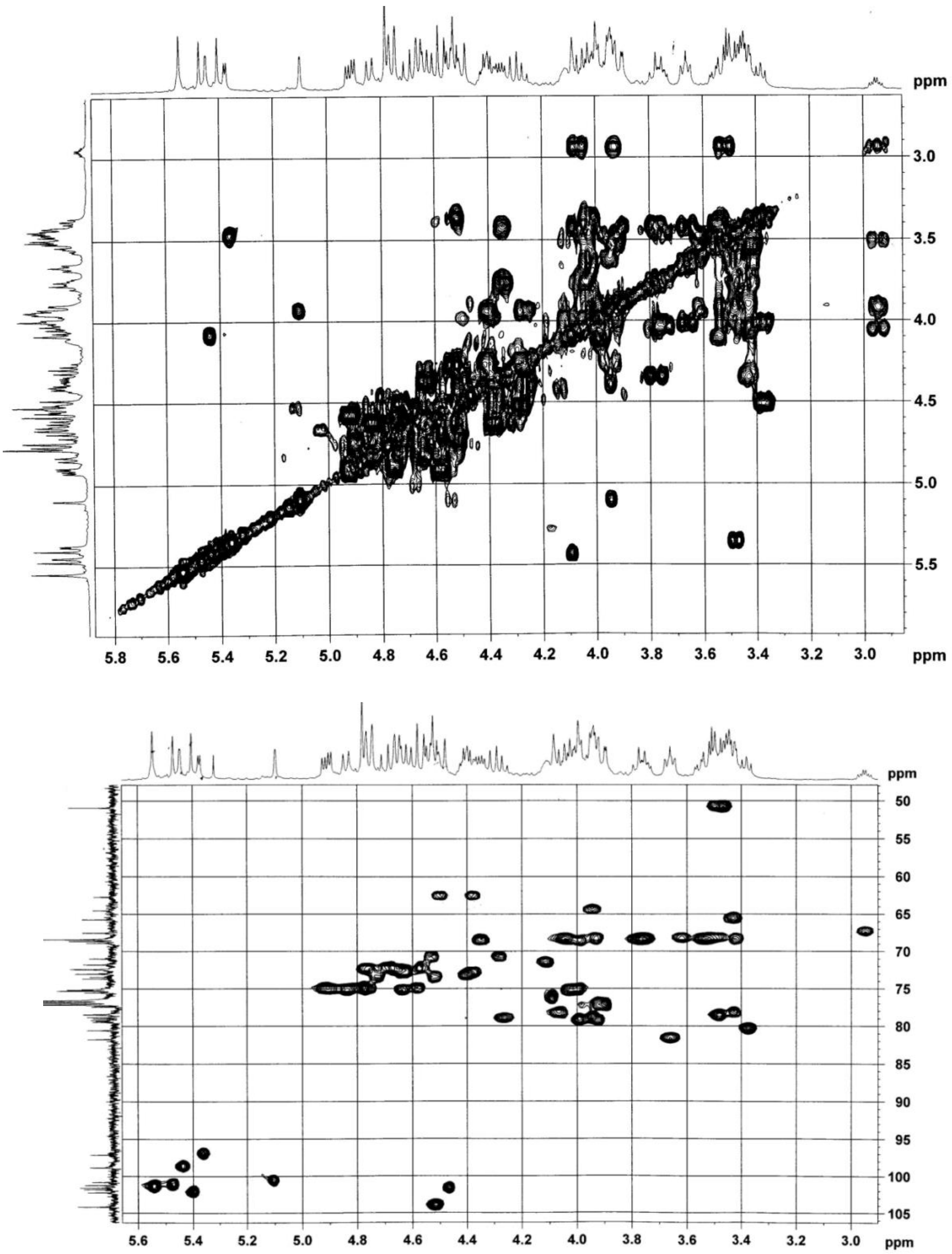

2D COSY and NMR spectra of 2-azidoethyl (2,3-di-*O*-benzyl-4,6-*O*-benzylidene-β-D-mannopyranosyl)-(1→4)-(6-*O*-benzoyl-2,3-di-*O*-benzyl-α-D-glucopyranosyl)-(1→3)-(2-*O*-benzyl-4,6-*O*-benzylidene-α-D-mannopyranosyl)-(1→2)-(3,4,6-tri-*O*-benzyl-α-D-mannopyranosyl)-(1→3)-2-*O*-benzyl-4,6-*O*-benzylidene-β-D-glucopyranoside (**20**) ($CDCl_3$) (selected region).

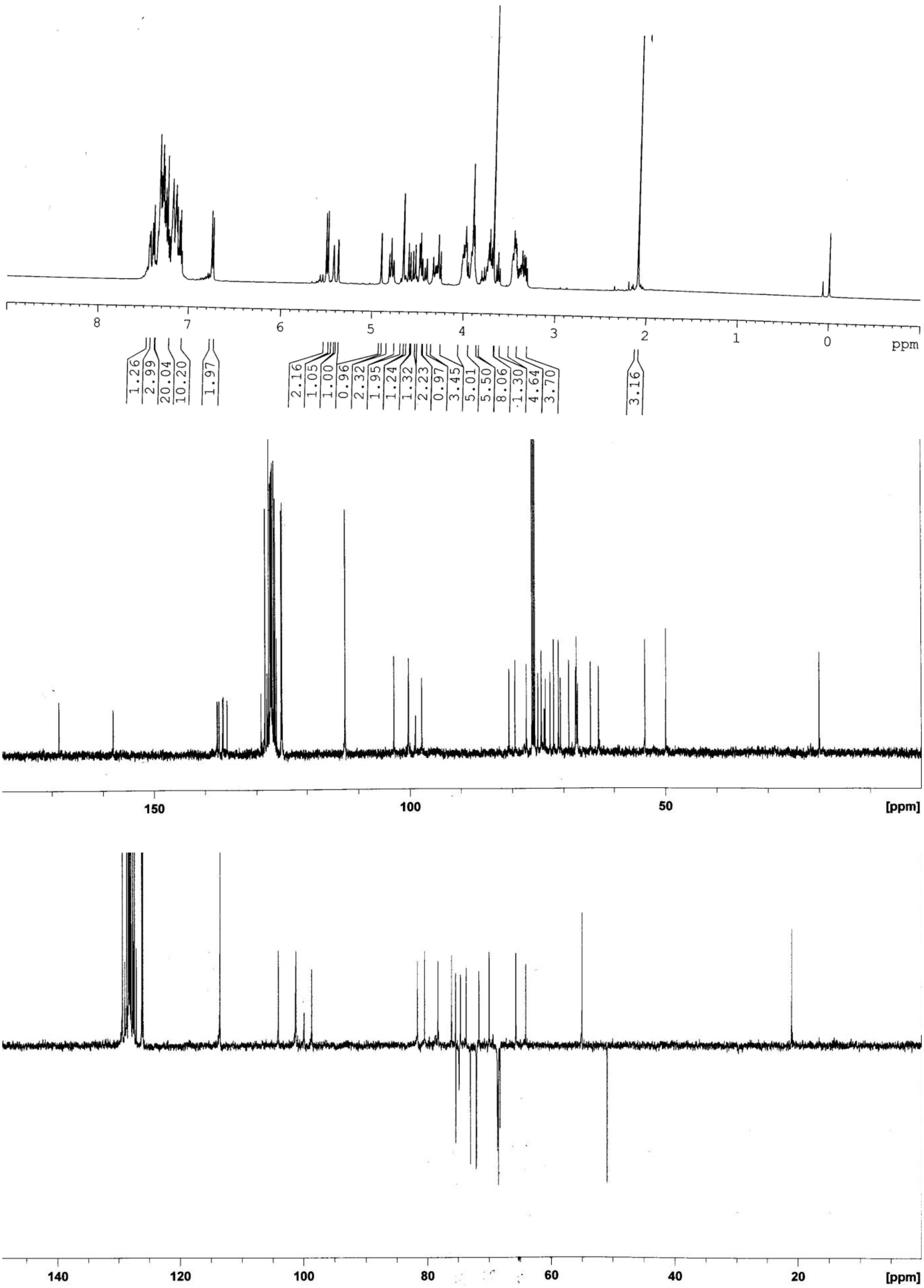

^1H and ^{13}C NMR spectra of 2-azidoethyl (2-*O*-acetyl-4,6-*O*-benzylidene-3-*O*-*p*-methoxybenzyl-α-D-mannopyranosyl)-(1→2)-(3,4,6-tri-*O*-benzyl-α-D-mannopyranosyl)-(1→3)-2-*O*-benzyl-4,6-*O*-benzylidene-β-D-glucopyranoside (**21**) ($CDCl_3$).

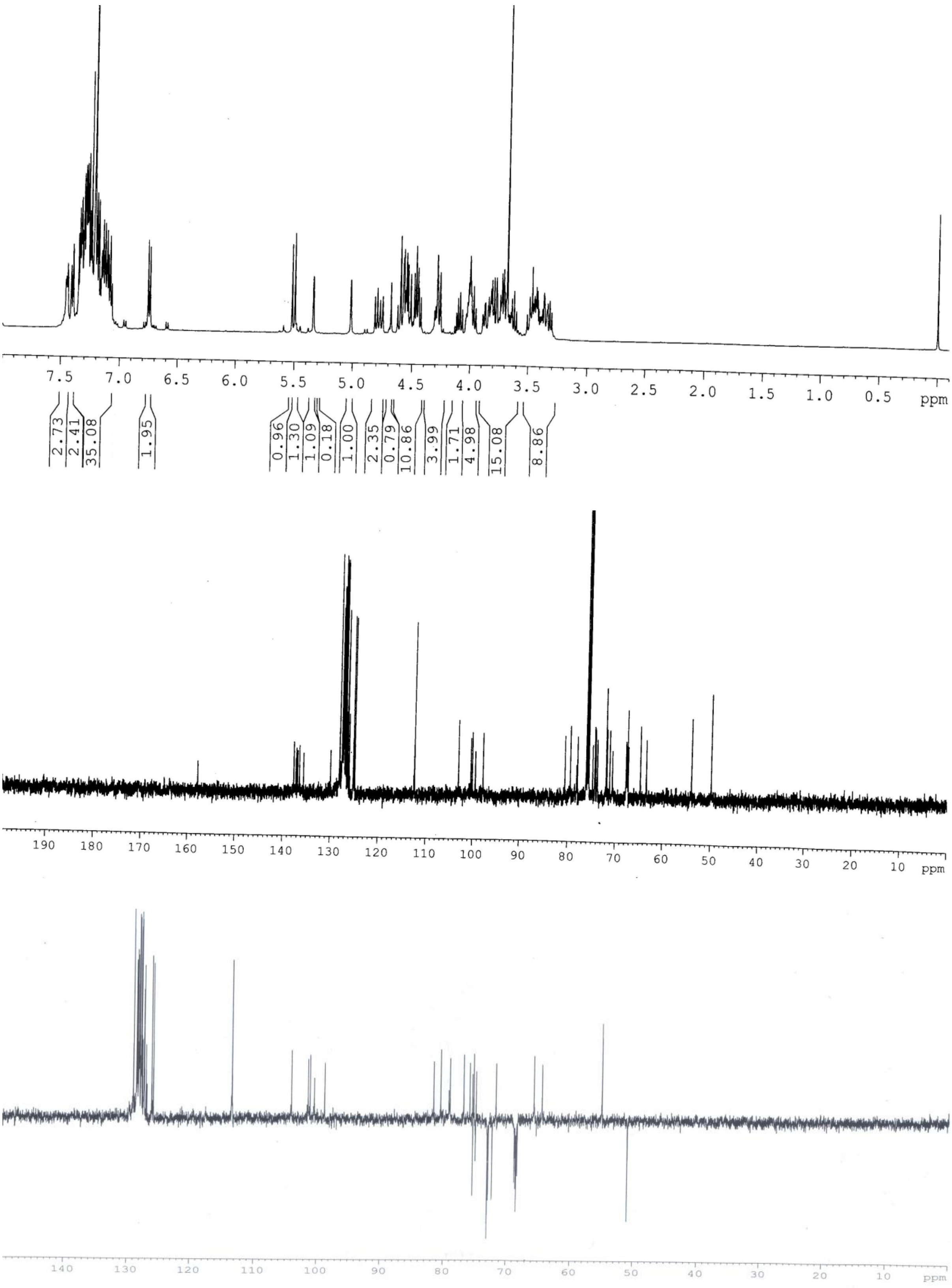

^{1}H and ^{13}C NMR spectra of 2-azidoethyl (2-*O*-benzyl-4,6-*O*-benzylidene-3-*O*-*p*-methoxybenzyl-α-D-mannopyranosyl)-(1→2)-(3,4,6-tri-*O*-benzyl-α-D-mannopyranosyl)-(1→3)-2-*O*-benzyl-4,6-*O*-benzylidene-β-D-glucopyranoside (**22**) ($CDCl_3$).

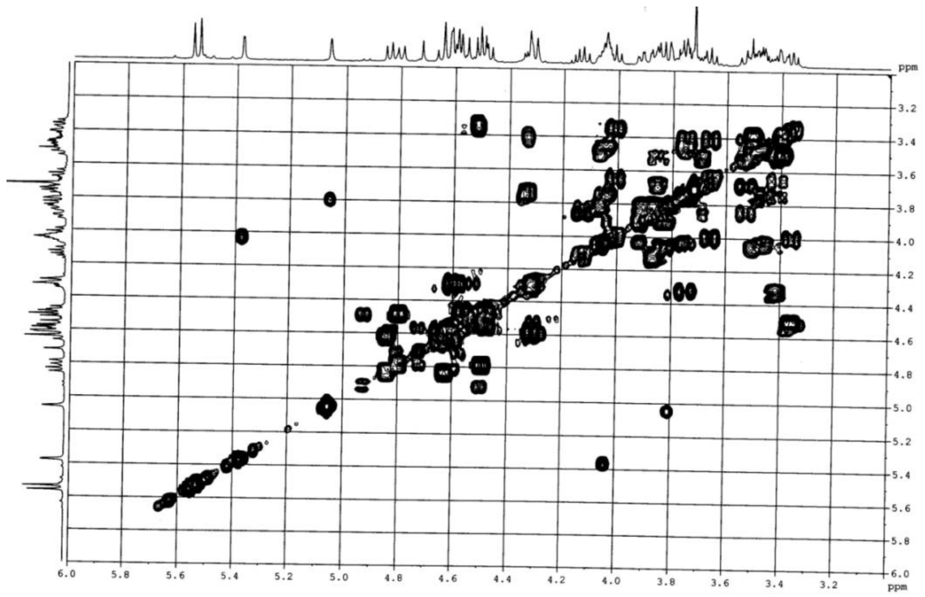

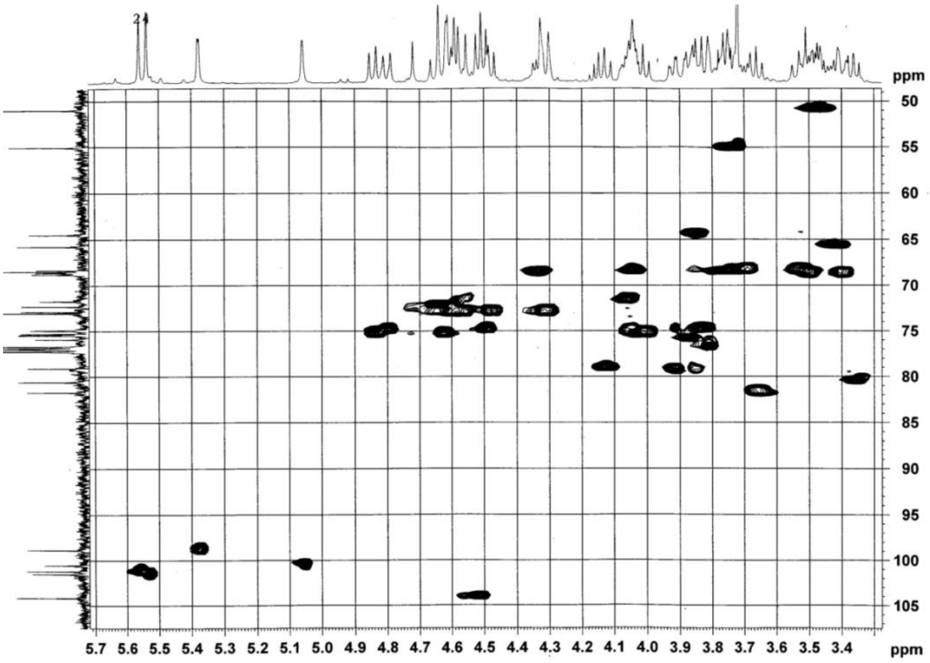

2D COSY and HSQC NMR spectra of 2-azidoethyl (2-*O*-benzyl-4,6-*O*-benzylidene-3-*O*-*p*-methoxybenzyl-α-D-mannopyranosyl)-(1→2)-(3,4,6-tri-*O*-benzyl-α-D-mannopyranosyl)-(1→3)-2-*O*-benzyl-4,6-*O*-benzylidene-β-D-glucopyranoside (**22**) ($CDCl_3$) (selected region).

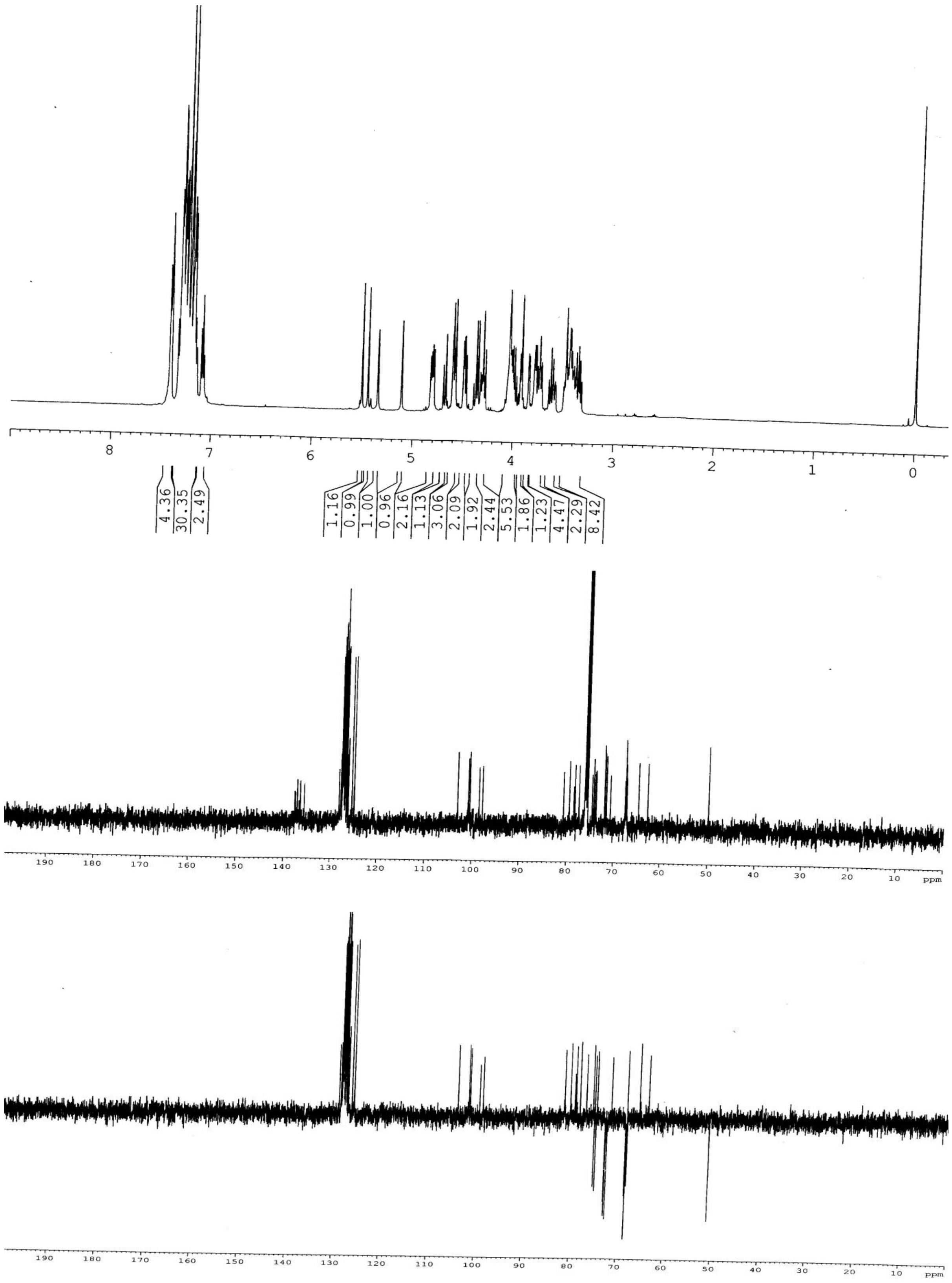

2-Azidoethyl (2-*O*-benzyl-4,6-*O*-benzylidene-α-D-mannopyranosyl)-(1→2)-(3,4,6-tri-*O*-benzyl-α-D-mannopyranosyl)-(1→3)-2-*O*-benzyl-4,6-*O*-benzylidene-β-D-glucopyranoside (**23**) ($CDCl_3$).
